# Combining DFT Calculations and Clustering Techniques to Screen Organic Monovalent Cations for Applications in Halide Perovskite Solar Cells

**DOI:** 10.1021/acsomega.6c03327

**Published:** 2026-06-05

**Authors:** Gabriel C. Bueno, Israel C. Ribeiro, Iván Ornelas-Cruz, Ronaldo C. Prati, Matheus P. Lima, Juarez L. F. Da Silva

**Affiliations:** † São Carlos Institute of Chemistry, University of São Paulo, 13560−970 São Carlos, São Paulo, Brazil; ‡ Center of Mathematics, Computation and Cognition, 28133 Federal University of ABC, 09210−580 Santo André, São Paulo, Brazil; ¶ Department of Physics, Federal University of São Carlos, 13565-905 São Carlos, São Paulo, Brazil

## Abstract

Surface passivation is widely used to improve the operational stability and efficiency of hybrid perovskite photovoltaics; however, the selection of passivation agents remains largely empirical. We present a computational framework that combines high-throughput density functional theory calculations with unsupervised clustering to analyze the adsorption of 134 organic cations, applied as post-treatment interfacial passivators, on zero-dimensional Cs_4_[PbI_6_] perovskite fragments. We find that passivation is not captured by a single descriptor (e.g., cation electron affinity or molecular geometry), but instead reflects a balance between interfacial electronic stabilization and structural deformation of the organic backbone. Rigid, highly π-conjugated cations exhibit large deformation penalties (up to 1.12 eV), which offset favorable interfacial interactions and weaken net adsorption. In contrast, bulky coordination-saturated cations are stabilized by dispersion-driven hydrophobic encapsulation and exhibit favorable deformation energies (down to −1.85 eV). Flexible primary amines and geometrically unhindered cations enable anchoring with near-zero deformation costs. Electronic-structure analysis indicates two regimes: conjugated molecules introduce low-lying states within the perovskite band gap (Type II alignment), which may act as charge-trapping states, whereas saturated backbones behave as wide-gap insulators (Type I alignment) that preserve the optoelectronic structure of the perovskite. Overall, the screening suggests that effective candidates should combine conformational flexibility (to limit deformation penalties) with electronic saturation (to reduce Type II trap formation) and thereby inform the design of stable perovskite interfaces for optoelectronic devices.

## Graphical abstract

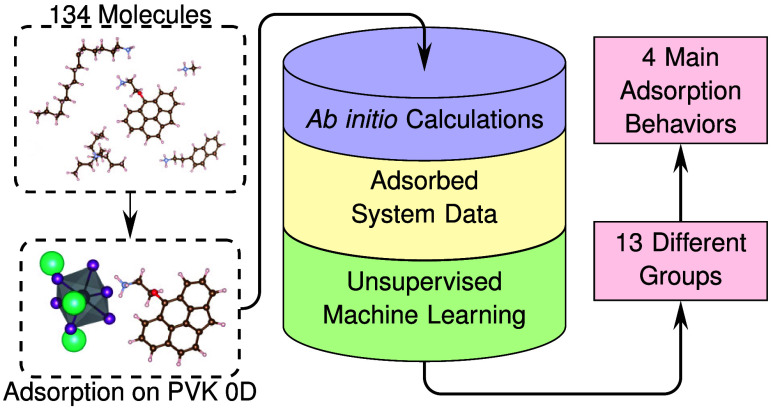

## 1 Introduction

Crystalline silicon devices remain the standard in photovoltaics, but they are constrained by the single-junction efficiency limit and high manufacturing costs. Rising demand for low-carbon energy motivates photovoltaic (PV) technologies that can deliver higher efficiencies at lower production costs.^1^ Metal-halide perovskite solar cells have reached power conversion efficiencies from 3.8 to 27.3% over 16 years.^2,3^ These gains arise from intrinsic optoelectronic properties, including long charge-carrier diffusion lengths, strong optical absorption, and chemically tunable band gaps.^4,5^ They are also compatible with low-temperature solution processing.^6^ Large-scale deployment remains limited by thermodynamic instability and sensitivity to moisture, elevated temperatures, and ultraviolet irradiation.^4,5^

This instability is commonly attributed to the high density of crystallographic defects in polycrystalline films, particularly at grain boundaries and heterointerfaces.^5,7,8^ Surface termination of the ABX_3_ perovskite lattice produces undercoordinated Pb^2+^ and I^–^ species (at the B and X sites, respectively). These sites are electronically active and can generate deep states within the band gap.^8,9^ Such defects impair device performance through two mechanisms: electronically, they act as nonradiative recombination centers that reduce *V*_OC_ and the fill factor; chemically, they promote hydration and facilitate ion migration, accelerating phase decomposition during operation.^8^ Mitigating surface and interfacial trap states is therefore essential for device performance and long-term stability.

Surface passivation is commonly accomplished through the deposition of organic modulators that coordinate with undercoordinated surface sites.^5,10^ Adsorption can suppress trap states by saturating dangling bonds and redistributing local charge density, while hydrophobic fragments reduce water exposure at reactive interfaces and impede moisture ingress.^8^ Although several functional groups, including amines, pyridines, and ammonium cations, have demonstrated passivation efficacy, discovery of new agents remains largely empirical and broadly generalizable design rules are still lacking.^5,10,11^

The microscopic parameters governing adsorption efficiency and passivation performance remain incompletely resolved.^6^ Enhanced Lewis basicity can strengthen interactions with undercoordinated Pb^2+^ sites; however, steric congestion can induce geometric rearrangements that increase deformation energies and destabilize the local structure. The energetic alignment between the molecular frontier orbitals and the edges of the perovskite band, essential for selective charge extraction and carrier blocking, is often assessed *a posteriori* rather than used as a predictive constraint.^4^ Evaluating this balance requires separating electronic bonding contributions from structural accommodation penalties of organic adsorbates.

In this work, we computationally screen 134 organic cations to identify the electronic descriptors that control bonding to undercoordinated perovskite-like motifs (e.g., Pb–I) and steric descriptors that govern structural accommodation at the interface. The molecules are treated as interfacial passivators (typically applied as post-treatments), rather than as species incorporated in significant concentrations at the *A* site. Thus, bulk perovskite metrics such as the Goldschmidt tolerance factor and the octahedral factor were not used as screening criteria for the organic cations. To make this screening tractable, we replace explicit periodic slab models with a simplified zero-dimensional (0D) Cs_4_[PbI_6_] fragment that preserves the relevant local chemistry while allowing high-throughput DFT calculations. We benchmark this 0D approach against previous periodic-slab simulations with 16 adsorbed molecules, which enables screening of a larger chemical space without sacrificing microscopic interpretability.

Finally, we apply unsupervised machine learning to separate the contributions of electronic stabilization (*E*_*int*_) and the structural cost of adsorption (*E*_def_). By comparing purely topological descriptors, namely the Many-Body Tensor Representation (MBTR), with explicit physicochemical features, we find that molecular geometry alone is insufficient to capture passivation behavior. Instead, the results indicate that passivation reflects the balance between orbital hybridization and the molecule’s conformational adaptability to the surface. This analysis provides mechanistic criteria for selecting interface modifiers in perovskite optoelectronic devices.

## 2 Theoretical Approach and Computational Details

### 2.1 Density Functional Theory

All first-principles calculations were performed using DFT, as implemented in the Fritz–Haber Institute *ab initio* molecular simulation package (FHI-aims).^12^ The Kohn–Sham equations (KS) were solved by employing numerical atom-centered orbitals (NAOs), which provide a high-accuracy representation of the electronic wave function while minimizing basis-set superposition error (BSSE).^12,13^ We adopted the standard *light-tier2* basis set (FHI-aims terminology), which augments the minimal free-atom basis with two additional tiers of polarization. This level of description balances computational efficiency and basis-set completeness, while providing a reliable description of diffuse electronic density at the organic–inorganic interface. Given the presence of heavy elements (Pb, I, Cs), scalar relativistic effects were included via the atomic Zero-Order Regular Approximation (ZORA) to describe core states and relativistic contraction effects required for heavy-metal halides.^14^ Spin–orbit coupling (SOC) was not included in the present workflow; therefore, absolute frontier-level positions of the Pb-halide fragment carry a quantitative SOC-related error (most prominently, a reduced inorganic gap and a shifted CBM). Because the inorganic fragment and computational settings are identical across the screened complexes, the SOC contribution is expected to be largely systematic for relative comparisons.

We used a dual-step computational protocol to separate structural relaxation from the prediction of electronic properties.

1. **Structural optimization:** All geometry optimizations were performed using the semilocal Perdew–Burke–Ernzerhof (PBE) formulation for the exchange-correlation energy functional.^15^ To account for noncovalent interactions relevant to adsorption, particularly London dispersion forces, we incorporated Grimme’s D3 dispersion correction scheme.^16^ In this approach, dispersion contributions are described through environment-dependent dispersion coefficients (*C*_6_) parametrized via coordination numbers, enabling a consistent description of long-range van der Waals interactions. Dispersion interactions are required to describe equilibrium adsorption geometries and separations at the organic–inorganic interface.
2. **Electronic structure refinement:** Given the known tendency of semilocal functionals such as PBE to underestimate fundamental band gaps in extended systems and frontier-orbital energy gaps in molecular species, and to induce spurious charge delocalization due to self-interaction error, final single-point energies were computed using the range-separated hybrid HSE06 functional.^17–19^ By incorporating 25% Hartree–Fock exchange in the short-range region, HSE06 improves the description of frontier-orbital energetics relative to semilocal functionals. Charge-transfer magnitudes (Δ*Q*) were evaluated using Hirshfeld population analysis and adopted as primary descriptors of passivation performance. These quantities were computed at the PBE level to balance computational accuracy and cost, while preserving relative charge-transfer trends across the screened molecular set.

The convergence of the Kohn–Sham (KS) equations was controlled through stringent self-consistency criteria. The self-consistent field (SCF) cycle was considered to converge when the total-energy change was below 1 × 10^–5^ eV, the charge-density variation below 1 × 10^–4^ *e*, and the change in the sum of eigenvalues below 1 × 10^–2^ eV. Geometry optimizations were performed until the maximum residual force on any atom was below 1 × 10^–2^ eV/Å.

### 2.2 Cluster Models and Chemical Space

#### 2.2.1 Selection of Organic Cations

Organic cations known to modulate the surface chemistry of metal-halide perovskites were considered, as they can passivate undercoordinated surface sites and stabilize interfacial structures in both two-dimensional (2D) and three-dimensional (3D) systems.^4,5,10^ In particular, molecules bearing ammonium, phosphonium, or sulfonium functional groups are suitable candidates because their positively charged headgroups establish strong electrostatic interactions with negatively charged sites on perovskite surfaces, whereas the alkyl or aromatic substituents allow systematic modulation of steric and electronic properties.^4,5,10,20,21^ Practical device implementation also depends on processability (e.g., solubility in polar aprotic solvents and the ability to form uniform post-treatment layers), which is not explicitly modeled in the present work.

Building on previous insights, the present computational screening broadens the explored chemical space beyond commonly studied short-chain alkylammonium cations by considering an extensive set of organic cations with systematic variations in alkyl chain length, degree of aromaticity, heteroatom incorporation, and halogen substitution patterns. The data set was assembled from the Open Perovskite Database, from which 134 organic cations were selected.^22^ This choice ensures that the screened structures have experimental precedent in the perovskite literature; nevertheless, practical accessibility can still vary (e.g., multistep syntheses, counterion dependence, and different levels of commercial availability). The structural files were then manually curated to ensure correct protonation states and optimized starting geometries, encompassing systematic variations in

1. **Head Groups:** Ammonium (−NH_3_^+^), formamidinium (−CH­(NH_2_)_2_^+^), phosphonium (–PH_3_^+^), and sulfonium (–SH_2_^+^).
2. **Tail Architecture:** Ranging from short alkyl chains to bulky polyaromatic systems (e.g., pyrene, fluorene derivatives).

The resulting chemical diversity enables a systematic evaluation of how subtle molecular modifications influence adsorption energetics in zero-dimensional perovskite clusters.

#### 2.2.2 The 0D Cs_4_[PbI_6_] Proxy Model

Explicit modeling of a large number of adsorbates on extended periodic slab models would entail a prohibitive computational cost. To address this limitation, we adopt a Cs_4_[PbI_6_] cluster proxy representing the fundamental structural unit of the perovskite lattice, namely an isolated [PbI_6_]^4–^ octahedron electrostatically stabilized by four equivalent Cs^+^ counterions.^10^ This 0D fragment serves as a surrogate for undercoordinated surface environments (e.g., vacancy sites and grain boundaries), where the translational symmetry of the bulk lattice is locally disrupted. Although the model does not describe band dispersion or long-range dielectric screening, it preserves the local electrostatic environment and orbital hybridization patterns governing adsorption energetics. Consequently, adsorption, interaction, and deformation energies are expected to be most transferable within this approximation, whereas absolute electronic level alignment should be interpreted qualitatively as an indicator of local in-gap state formation. This simplification enables large-scale screening of adsorbate interactions while retaining an accurate description of local chemical bonding. As detailed in the Supporting Information (SI), for a set of 16 representative molecules, the adsorption and interaction energies predicted by the cluster model differed by approximately 0.19 eV from those obtained with periodic two-layer slab calculations, confirming that the fragment model reliably captures local adsorption energies.^5^

#### 2.2.3 Generation of the Passivated Configurations

The passivated complexes were generated through a substitution protocol in which a Cs atom of the reference cluster was replaced by the organic cation. The molecules were initially oriented so that the anchoring functional group faced the central [PbI_6_] octahedron, favoring electrostatic interaction with the inorganic fragment. To ensure a consistent starting geometry throughout the data set, the separation between the donor heavy atom and the Pb atom (*d*_donor–Pb_) was fixed at 4.0 Å prior to complete structural relaxation. A schematic representation of the procedure is shown in Figure 1.

**Figure 1:**
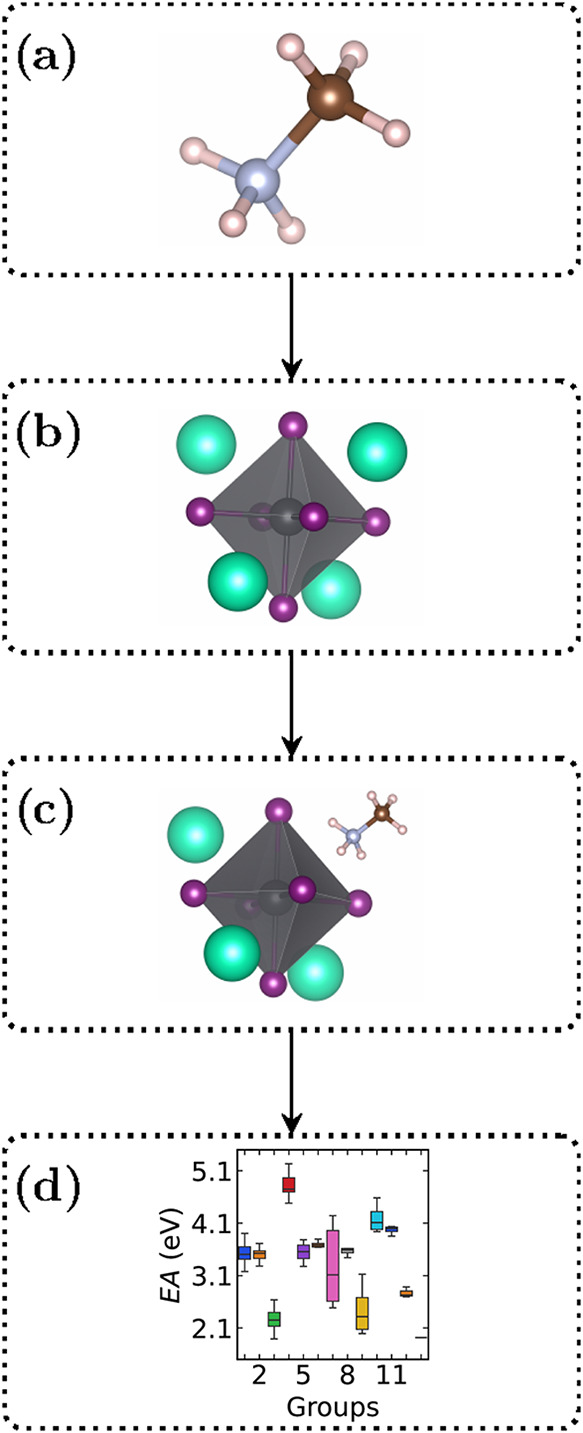
Flowchart of the methodology used to construct 134 initial structures of organic adsorbates on 0D perovskite fragments. The steps are (a) DFT-PBE+D3 optimization of the organic molecule; (b) DFT-PBE+D3 optimization of the 0D fragment; (c) substitution of one Cs with the organic molecule, followed by DFT-PBE+D3 optimization of the hybrid 0D perovskite; and (d) single-point DFT-HSE06 calculations on the final structures to better describe electronic and energetic properties, followed by Agglomerative Hierarchical Clustering (HC), yielding 13 groups.

### 2.3 Clustering and Data Analysis

#### 2.3.1 Goal and General Workflow

To analyze the passivation behavior of the screened organic cations, we used an unsupervised machine-learning approach to partition the data set into chemically interpretable groups, which were then examined in terms of adsorption energetics and electronic-structure descriptors. The workflow consisted of: (i) constructing a feature matrix encompassing compositional, structural, electronic, and energetic descriptors; (ii) performing feature scaling to ensure consistency of the distance metric; (iii) applying agglomerative hierarchical clustering to identify groups of similar molecular structures; and (iv) quantitatively assessing the quality of the resulting partitions using two global clustering metrics based on the analysis of variance within each group. All preprocessing and clustering procedures were performed with scikit-learn.^23^

#### 2.3.2 Descriptor Set and Normalization

Each molecule *i* was represented by a characteristic vector **x** ^*i*^ = (*x*_1_^*i*^,···, *x*_*j*_^*i*^,···,*x*_*F*_^*i*^), where *F* represents the number of features; combining the following features:

1. Compositional descriptors: counts of each element and identity of the donor atom closest to the center Pb, e.g., N, P, S.
2. Structural descriptors: octahedral volume (*V*_oct_), donor-lead distance (*d*_donor–Pb_), molecular mean radius (*r*_mol_), and molecular volume (*V*_mol_).
3. Electronic descriptors for both the passivated 0D perovskite proxy model (mol/PVK) and the isolated molecule (mol): frontier orbital energies (*E*_HOMO_^mol/PVK^, *E*_LUMO_^mol/PVK^, *E*_HOMO_^mol^, and *E*_LUMO_^mol^) and gaps (*E*_*g*_^mol/PVK^ and *E*_*g*_^mol^).
4. Energetic and charge-transfer descriptors: adsorption energy (*E*_ad_), interaction energy (*E*_int_), deformation energy (*E*_def_), electron affinity (*EA*), and transferred charge (Δ*Q*).

Because distance-based clustering (Euclidean metric) and Ward linkage are scale sensitive, all continuous descriptors were normalized using Min-Max scaling to map each feature to [0,1].^23^

1 $$x_{j}^{i‐\mathrm{norm}}=\frac{x_{j}^{i}-x_{j}^{\min}}{x_{j}^{\max}-x_{j}^{\min}}$$

where *x*_*j*_^min^ and *x*_*j*_^max^ are the minimum and maximum of the feature *j* across the entire data set.

#### 2.3.3 Variance-Based Cluster Quality Metrics

To evaluate how tight and how balanced the resulting groups are across the full descriptor space, we defined two global metrics based on intragroup dispersion. For a given group *g* (with *N*_*g*_ members) and feature *j*, the intragroup standard deviation is

2 $$\sigma_{g,j}=\sqrt{\frac{1}{N_{g}-1}\sum_{i=1}^{N_{g}}{\left(x_{j}^{i‐\mathrm{norm}}-\mu_{g,j}\right)}^{2}}$$

where μ_*g*,*j*_ is the mean of the characteristic *j* within the group *g*. The mean dispersion of the feature *j* across the *G* groups is then

3 $$S_{j}=\frac{1}{G}\sum_{g=1}^{G}\sigma_{g,j}$$

We define global cohesion as the average intragroup spread over all *F* features,

4 $$C_{\mathrm{global}}=\frac{1}{F}\sum_{j=1}^{F}S_{j}$$

so that smaller *C*_global_ indicates, on average, denser groups in the normalized descriptor space. However, a low mean spread can be misleading if group quality is driven by only a subset of descriptors (e.g., structural attributes), while others remain highly dispersed (e.g., electronic descriptors). To quantify this balance, we define *Global Uniformity* as the dispersion of the *S*_*j*_ values around *C*_global_:

5 $$U_{\mathrm{global}}=\sqrt{\frac{1}{F-1}\sum_{j=1}^{F}{\left(S_{j}-C_{\mathrm{global}}\right)}^{2}}$$

Therefore, a useful partition simultaneously minimizes *C*_global_ (compactness) and *U*_global_ (descriptor-balance), which is important here because adsorption performance is multifactorial (electronic stabilization vs structural accommodation).

#### 2.3.4 Clustering Algorithm and Selection of the Number of Groups

We employ agglomerative hierarchical clustering with Ward’s linkage, which iteratively merges groups to minimize the increase in within-group sum of squares (i.e., total intragroup variance).^23,24^ Ward linkage is naturally paired with the Euclidean metric, providing a consistent geometry for the scaled feature vectors and favoring partitions that are variance-optimal in the descriptor space.

#### 2.3.5 Data Set Representations: MBTR vs Physicochemical Feature Vectors

We compared two molecular representations to assess how much of the passivation behavior is encoded in structure alone vs the structure–property response. MBTR maps each structure to a continuous descriptor by broadening the distributions of atomic identities, interatomic distances, and angles (orders *k* = 1, 2, 3).^25,26^ Because MBTR produces high-dimensional vectors that can contain redundant topological information, we applied Principal Component Analysis (PCA) and retained the number of components required to reproduce 98% of the variance.^23^ The physicochemical representation uses the explicit compositional, structural, electronic, and energetic descriptors listed above (Section 2.3), which encode the response of the interaction between the molecule and the 0D perovskite proxy model. These descriptors capture the trade-off that governs adsorption-driven passivation: balancing electronic stabilization (*E*_int_) with the structural penalty of adsorbate accommodation (*E*_def_).

#### 2.3.6 Clustering Protocols (Three Complementary Tests)

To separate the role of topology, adsorption-induced distortion, and functional interface descriptors, we performed three clustering protocols:

- **Molecules MBTR (gas-phase molecule geometry).** MBTR descriptors were computed for organic cations in their optimized gas-phase geometries to test whether intrinsic molecular topology alone differentiates passivators without explicitly encoding the adsorption environment.
- **Molecule/PVK MBTR (adsorbed geometry).** MBTR descriptors were computed for organic cations in their relaxed adsorbed geometries on the perovskite fragment. This includes adsorption-induced distortions and orientations and tests whether binding-related geometry changes improve the relevance of topology-based groups.
- **Physicochemical features (molecule/PVK response).** Clustering used explicit descriptor vectors from molecule/PVK calculations, grouping molecules by their computed passivation behavior and separating them according to the coupled electronic–steric balance governing adsorption and defect passivation.

## 3 Results and Discussion

We first define an optimal passivation agent. In this work, effective passivation is treated as a multiobjective optimization problem that requires satisfying three concurrent criteria: (i) energetic stability, characterized by large negative adsorption energies (*E*_ad_); (ii) mechanical compatibility, indicated by near-zero or negative deformation energies (*E*_def_) that denote strain-free accommodation; and (iii) electronic suitability, defined by a Type I energy-level alignment that preserves the perovskite fundamental gap without introducing deep trap states. The following sections evaluate the data set against these combined metrics.

The results are divided into three sections. The first focuses on structural analysis, including chemical categorization of the organic molecules. The second covers electronic analysis, correlating frontier orbital energies with the electronic gaps of perovskite fragments. The third presents an energetic analysis, examining key physicochemical descriptors.

### 3.1 Data set Characterization via Clustering Techniques

#### 3.1.1 Selection of Clustering Granularity (*G*) and Quantitative Comparison of Representations

To select a clustering granularity that preserves chemical resolution without overpartitioning, we tested several dendrogram cuts (*G* = 9, 11, 13) using the two variance-based metrics defined above: Global Cohesion (*C*_global_), the average intragroup dispersion across all descriptors, and Global Uniformity (*U*_global_), which quantifies how evenly this cohesion is distributed across descriptors. A physically meaningful partition should therefore minimize both metrics and produce clusters that remain compact across representations, from geometric to full physicochemical descriptors.

At *G* = 13, the two representations based on MBTR (Molecules MBTR and Molecule/PVK MBTR) yielded essentially identical clustering quality, with the same global dispersion metrics (*C*_global_ = 0.07, *U*_global_ = 0.06) and nearly identical spreads within the group for each property block. Adsorption-induced geometric distortions therefore do not significantly alter the dominant topological fingerprints captured by MBTR at the clustering-relevant level. MBTR-based clustering yields groups with comparatively large intragroup dispersion in electronic and energetic descriptor blocks (both 0.12–0.13; Table 1), indicating that molecules grouped by topology can still differ significantly in frontier-level alignment, charge transfer, and binding energetics. This reflects the fact that adsorption-driven passivation is governed by local donor–Pb interactions and charge rearrangement, which are not uniquely determined by global molecular topology.

**Table 1:** Intra-Group Consistency Assessment of Structural and Physicochemical Representations after Min-Max Normalization for *G* = 13^a^

property group	molecule/PVK MBTR	molecules MBTR	physical chemistry
composition	0.02	0.02	0.02
electronic	0.12	0.12	0.08
energetic	0.13	0.13	0.08
structural	0.10	0.10	0.09
*C*_global_	0.07	0.07	0.05
*U*_global_	0.06	0.06	0.04

a Metrics include mean standard deviations for property groups, Global Cohesion (*C*_global_), and Global Uniformity (*U*_global_).

In contrast, clustering based on physicochemical feature vectors yields a tighter and more balanced partition at *G* = 13, as reflected by lower global metrics (*C*_global_ = 0.05 and *U*_global_ = 0.04; Table 1) and reduced intragroup dispersion in the electronic and energetic descriptor blocks (both 0.08). These descriptors explicitly capture the molecule/PVK interaction, including electronic stabilization (e.g., *E*_int_, Δ*Q*, frontier-level alignment) and the structural cost of adsorption (e.g., *E*_def_, *d*_donor–Pb_, *V*_oct_). As a result, molecules are grouped according to similar binding behavior and comparable electronic and energetic responses, rather than shared topology alone. The lower *U*_global_ further indicates that this compactness is achieved consistently across descriptor categories, reflecting a balanced, multidescriptor similarity aligned with the multifactorial nature of passivation.

#### 3.1.2 Justification for the Final Choice *G* = 13 and Representation Dependence

The PCA maps in Figure 2 support selecting *G* = 13 as the final clustering solution. Panels A (Molecule/PVK MBTR) and B (Molecules MBTR) are nearly identical, indicating that adsorption-induced distortions on the perovskite fragment do not substantially modify the dominant MBTR fingerprints. As a result, MBTR-based clustering shows limited discriminative power at higher chemical diversity, grouping a large fraction of structures into a single dominant cluster (Cluster 1, ∼64% of the data set; 86 molecules), which mixes chemically distinct species such as *sp* ^3^ linear primary amines and sp^2^ aromatic cations (Figure 2A,B).

**Figure 2:**
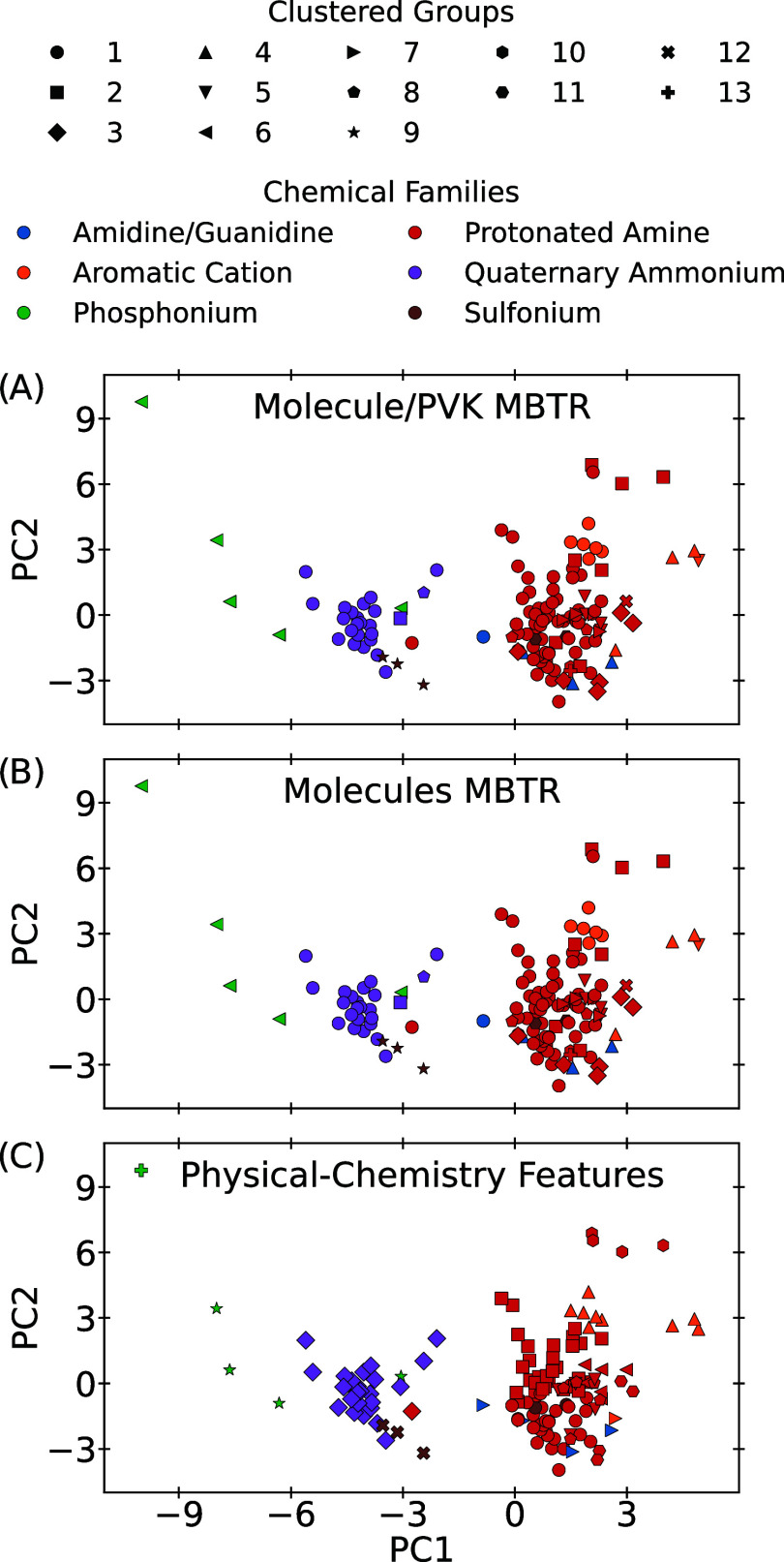
PCA projection and Agglomerative Hierarchical Clustering of the data set. All PCA spaces were obtained by reducing the same high-dimensional physicochemical descriptor vector. (A) Projection using the Molecule/PVK MBTR representation (organic cations in relaxed geometries on the perovskite surface). (B) Projection using the Molecules MBTR representation (optimized gas-phase molecules). (C) Projection using explicit physicochemical feature vectors. Marker colors indicate chemical families identified by SMILES pattern matching, and marker shapes indicate HC groups (*G* = 13), ordered by decreasing population from Group 1 (most populated) to Group 13 (least populated).

In contrast, the physicochemical representation (Figure 2C) produces a tighter and more interpretable separation by encoding the adsorption response, including electronic stabilization (*E*_int_, Δ*Q*, frontier-level alignment) and structural accommodation costs (*E*_def_ and geometric descriptors). In this response-sensitive space, selecting *G* = 13 increases separation between adsorption motifs while preserving coherence in passivation-relevant descriptors, consistent with the lower values *C*_global_ and *U*_global_.

#### 3.1.3 Chemical Interpretability and Independent Validation by SMILES/RDKit Fingerprinting

To evaluate whether the optimized clusters correspond to chemically meaningful families, we classified cations using SMILES strings and RDKit-based substructure matching and applied a hierarchical scheme to assign each molecule to an intuitive class (e.g., protonated amines, quaternary ammoniums, aromatic cations).^27,28^ This independent labeling enables a direct comparison between unsupervised clusters (marker symbols, Groups 1–13) and molecular identities (colors) in Figure 2. At *G* = 13, physicochemical clustering shows the highest chemical interpretability. Primary/protonated amines are distributed across multiple groups (Groups 1, 2, 5, 6, 10, 11), consistent with different adsorption responses (e.g., different balances between *E*_int_ and *E*_def_ and variations in frontier-level alignment). In contrast, quaternary ammoniums form a distinct group (Group 3), and resonance-stabilized families are separated (pyridinium in Group 4, amidinium derivatives in Group 7). These results indicate that structure-only clustering (MBTR) does not resolve key structure–property relationships, whereas physicochemical representation yields a chemically interpretable partition at *G* = 13 that supports identification and ranking of passivation candidates.

#### 3.1.4 Representative Members of the *G* = 13 Clusters and Coordination Motifs

Figure 3 displays one representative adsorbate from each of the *G* = 13 clusters bound to the [Cs_3_(PbI_6_)]^−^ fragment, thereby summarizing the coordination motifs identified in the final clustering. The selected molecules encompass a broad range of donor chemistries, dominated by N-centered cations but also including P- and S-based species, and they span diverse steric profiles, from compact aliphatic substituents to extended, rigid aromatic scaffolds. The donor–Pb separations reported in each panel (3.90–5.94 Å) differentiate adsorption geometries according to the strength and nature of the interaction. Shorter distances (∼4.0 Å) correspond to configurations that enable enhanced orbital overlap and electronic stabilization, whereas larger separations (∼5.5 Å) arise from steric congestion and/or backbone rigidity that impede close approach, resulting in increased structural distortion and diminished orbital hybridization. The representatives in Figure 3 thus associate each cluster with a characteristic coordination motif and steric regime, thereby establishing a direct structure–function relationship between molecular architecture and passivation behavior.

**Figure 3:**
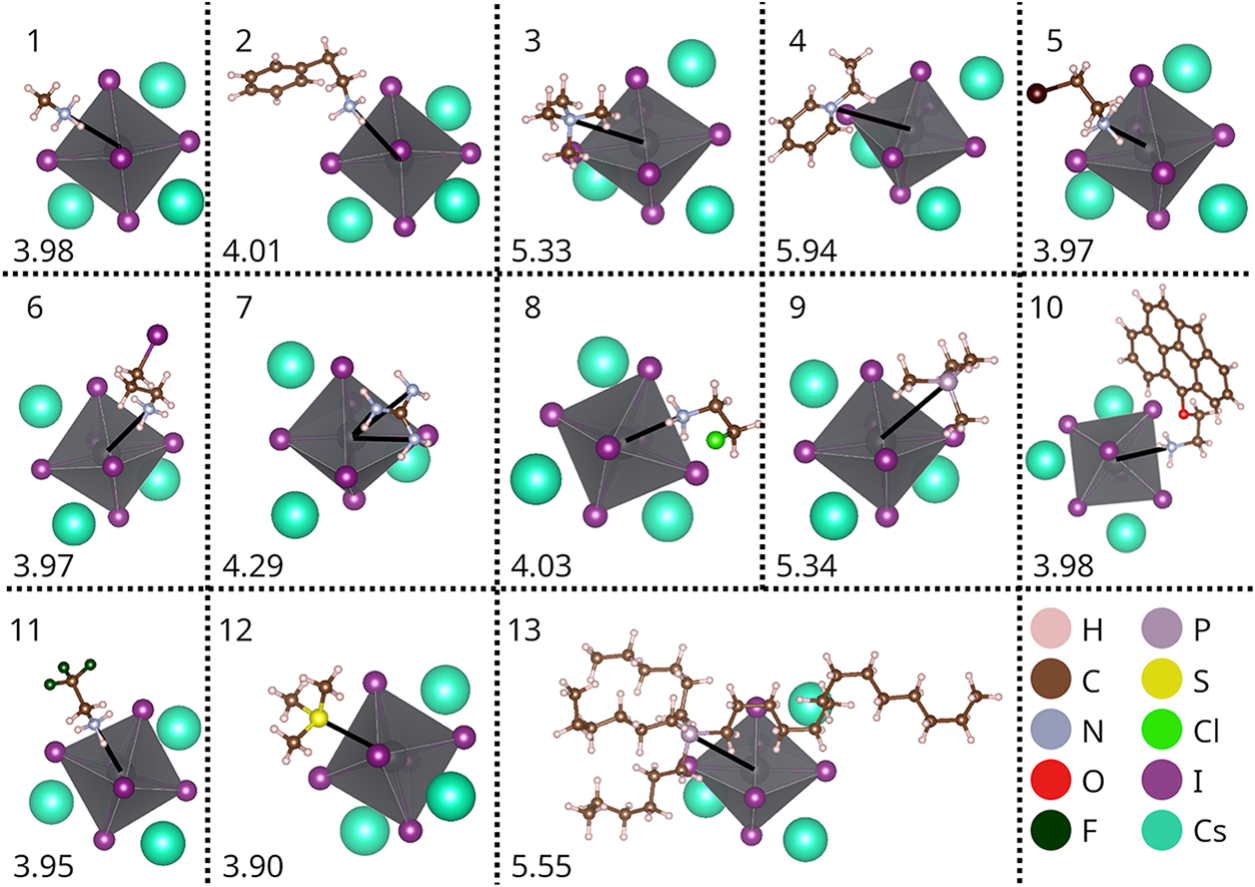
Chemical structures of the 13 representative molecules, selected from each cluster obtained via hierarchical clustering (HC) based on physicochemical descriptors. The donor-to-lead distances (*d*_donor–Pb_) in Å, represented by the black lines between the organic molecules and the PVK fragments, are indicated below each structure. The groups are organized from left to right and top to bottom in ascending order: Group 1 (methylammonium), Group 2 (phenylethylammonium), Group 3 (tetramethylammonium), Group 4 (N-ethylpyridinium), Group 5 (2-bromoethylammonium), Group 6 (3-iodopropylammonium), Group 7 (guanidinium), Group 8 (2-chloroethylammonium), Group 9 (tetramethylphosphonium), Group 10 (pyrene-O-ethylammonium), Group 11 (2,2,2-trifluoroethylammonium), Group 12 (trimethylsulfonium), and Group 13 (trihexyltetradecylphosphonium).

### 3.2 Structural Properties

HC based on physicochemical descriptors was further analyzed in terms of five structural quantities, as shown in Figure 4: octahedral volume (*V*_oct_), donor–Pb distance (*d*_donor–Pb_), average molecular radius (*r*_mol_), molecular volume (*V*_mol_), and number of atoms (*n*_atoms_). Correlating their statistical distributions with the chemical classification indicates a consistent structure–property relationship. The 13 groups can be merged into three main structural macro-families that differ in their statistical signatures and chemical identity.

1. **Rigid/conjugated rings (Groups 4 and 7):** Distinguished by donor atoms directly incorporated into rigid, π-conjugated structures, such as pyridiniums, triazoliums, and formamidiniums.
2. **Coordination-saturated donors (Groups 3, 9, and 13):** Characterized by highly coordinated cationic centers, predominantly involving quaternized nitrogen (N) and phosphorus (P) atoms (e.g., tetraalkylammoniums and tetraalkylphosphoniums).
3. **Flexible chains and primary amines (Groups 1, 2, 5, 6, 8, 10, 11, and 12):** The most diverse macro-group, dominated by primary amine groups attached to organic chains of varying length and composition, alongside cations such as sulfoniums (Group 12). These range from small saturated chains (Group 1) and halogenated chains (Groups 5, 6, 8, 11), to moderately sized hybrid aromatic amines (Group 2) and large hydrophobic groups such as pyrene derivatives (Group 10).

**Figure 4:**
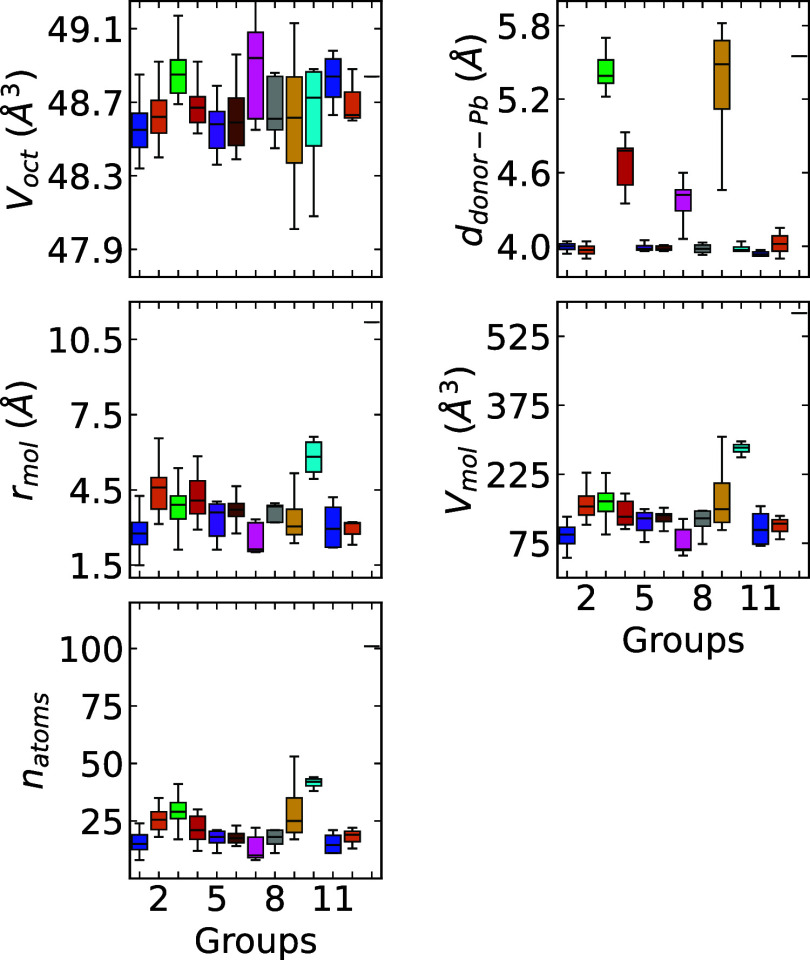
Comparative distribution of structural descriptors across the 13 groups. Individual data points with overlaid boxplots show dispersion and central tendency in each group. The structural parameters are iodine octahedra volume (*V*_oct_), the distance between the nearest donor atom to the Pb center (*d*_donor–Pb_), the average radius of the organic molecule (*r*_mol_), the organic molecule volume (*V*_mol_), and the number of atoms in the organic molecule (*n*_atoms_), characterizing system geometry.

#### 3.2.1 Nearest Donor-to-Lead Distance: Role of Steric Hindrance

The parameter *d*_donor–Pb_ served as the primary descriptor for distinguishing between the different families, with the observed trends predominantly governed by steric demands of the respective chemical species.

1. **High values (Groups 3, 9, and 13):** These groups contain quaternized donors and exhibit the highest median distances (5.31–5.55 Å). Chemically, this behavior is associated with highly coordinated cationic centers. These bulky, multicoordinated centers introduce significant steric hindrance and prevent the organic molecule from approaching the lead atom closely.
2. **Intermediate values (Groups 4 and 7):** These groups present an intermediate regime, with median distances of approximately 4.36–4.80 Å. This behavior originates from rigid nitrogen-based π-conjugated systems. Although these planar moieties impose defined spatial constraints, their steric effects are less pronounced than those of quaternized cations, yet are more restrictive than the conformational flexibility of primary amines.
3. **Low values (Groups 1, 2, 5, 6, 8, 10, 11, and 12):** These subpopulations are characterized by the shortest distances, tightly grouped within 3.94–4.02 Å. Chemically, these groups are composed predominantly of flexible chains with a terminal primary amine acting as the donor. This conformational flexibility allows the molecules to adopt favorable geometries and facilitates closer approach and interaction with the lead center, regardless of the bulkiness of the remaining organic tail.

#### 3.2.2 Size and shape of the organic molecules: mapping chemical volume to group identity

The variability in *n*_atoms_ and *V*_mol_ can be associated with the molecular characteristics of the identified subgroups.

1. **Moderately sized, rigid donor systems (Groups 4 and 7):** Despite having a relatively small to moderate number of atoms (∼11–21), the planarity of the functional rings favors a defined orientation of the molecule toward the surface, consistent with the intermediate dispersion observed in *d*_donor–Pb_.
2. **Coordination-Saturated Donors (Groups 3, 9, and 13):** Excluding extreme cases such as Group 13, which includes large aliphatic chains exceeding 100 atoms, Groups 3 and 9 exhibit atom counts comparable to other groups (∼29–31). However, the central donor site is effectively shielded, limiting its approach to the Pb center (∼5.3 Å).
3. **Molecule volume (** *V*_mol_ **) in flexible chains and primary amines:** The subdivision of the primary-amine groups (Groups 1, 2, 5, 6, 8, 10, 11, and 12) is primarily associated with differences in molecular volume and composition, rather than variations in donor–Pb distances:
  (a) *Small molecules (Groups 1, 5, 6, 8, and 11):* These groups exhibit the lowest atom counts (∼11–24) and the smallest molecular volumes (<130 Å^3^), corresponding predominantly to short saturated chains and monohalogenated species.
  (b) *Aromatic–amine hybrids (Group 2):* This group shows moderately higher atom counts (∼28) and intermediate volumes (∼165 Å^3^), corresponding to functionalized aromatic systems connected to the PVK through a flexible saturated amine linker.
  (c) *Large molecules (Group 10):* This group includes highly voluminous hydrophobic structures, such as pyrene derivatives, characterized by larger atom counts (∼42) and molecular volumes (∼281 Å^3^). Despite their steric bulk, the presence of a flexible aliphatic spacer allows the amine group to reach *d*_donor–Pb_ values comparable to those of smaller molecules.

#### 3.2.3 Octahedral Volume: Stability of the Inorganic Core

The parameter *V*_oct_ provides a metric for assessing the structural stability of the system. Despite variations in the size, volume, and rigidity of organic adsorbates, the volume of the iodine octahedron remains conserved across all 13 groups, within 48.51–48.89 Å^3^. This limited variation indicates that adsorption does not induce appreciable distortion of the [PbI_6_]^4–^ octahedron. Irrespective of whether the organic overlayer corresponds to a phosphonium cation (Group 13), a rigid pyridinium (Group 4), or a small flexible aliphatic amine (Group 1), the inorganic core preserves its geometric integrity. As reference, in α-APbI_3_ perovskites (with A = Cs, MA, or FA), *V*_oct_ ranges from 41–43 Å^3^.^29–31^ The larger values obtained in this work may be associated with the nonperiodic nature of the PVK model.

### 3.3 Electronic Characterization

We analyze the influence of organic passivation on the electronic properties of the perovskite surface by comparing the electronic structure across the identified groups. Figure 5 correlates the intrinsic frontier orbitals of the isolated organic molecules with the electronic configuration of the passivated perovskite fragments. In the nonperiodic cluster framework, orbital energies are referenced to a vacuum level defined by the asymptotic electrostatic potential far from the finite fragment, enabling consistent *relative* comparisons between the pristine inorganic cluster, the isolated molecule, and the adsorbed complex within the same setup. Kohn–Sham eigenvalues are method dependent and are not quasiparticle energies; therefore, the electronic descriptors discussed below are used to identify qualitative trends (e.g., the emergence of localized in-gap states upon adsorption), rather than quantitative bulk band offsets. The left panels show variations in the lowest unoccupied molecular orbital (*E*_LUMO_^mol/*PVK*^), the highest occupied molecular orbital (*E*_HOMO_^mol/PVK^), and the energy gap (*E*_*g*_^mol/PVK^) of the hybrid system, while the right panels show the corresponding intrinsic quantities of the organic adsorbates (*E*_LUMO_^mol^, *E*_HOMO_^mol^, and *E*_*g*_^mol^). This visualization allows evaluation of how the electronic character of the passivating layer (insulating, π-conjugated, or predominantly electrostatic) affects energy-level alignment and the emergence of trap states.

**Figure 5:**
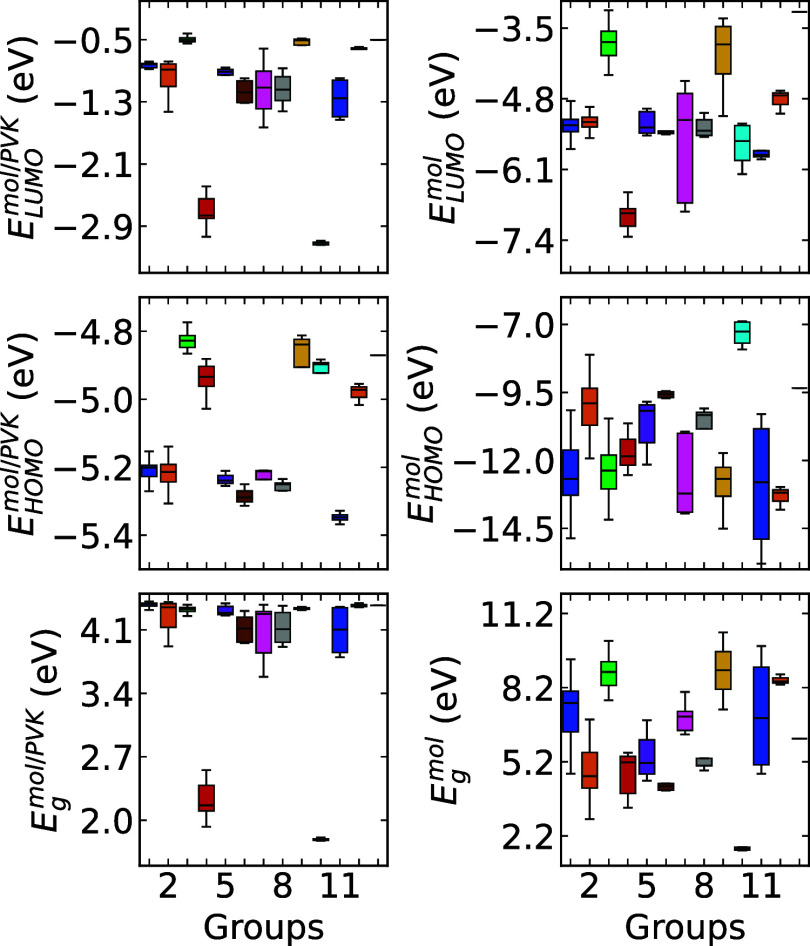
Comparative distribution of electronic descriptors obtained via DFT calculations using the hybrid functional HSE06 across the HC groups. The left panels show the electronic properties of the passivated perovskites: *E*_LUMO_^mol/PVK^, *E*_HOMO_^mol/PVK^, and the energy gap *E*_g_^mol/PVK^. The right panels show the properties of the isolated gas-phase organic molecules: *E*_LUMO_^mol^, *E*_HOMO_^mol^, and *E*_g_^mol^. All energies are given in eV.

#### 3.3.1 Highest Occupied Molecular Orbital

The valence-band edge is primarily governed by electrostatic effects rather than direct orbital mixing.

1. **Electrostatic destabilization (Groups 3, 4, 9, and 10):** The groups that encompass coordination-saturated donors, such as quaternary ammoniums (Group 3) and phosphoniums (Group 9), as well as highly conjugated systems (Groups 4 and 10), exhibit the highest (least negative) HOMO values, with average values between −4.93 and –4.83 eV. This destabilization is driven by surface dipoles and localized positive charges associated with bulky saturated cations, or by electron-rich π systems near the interface, which shift the local vacuum level and raise the HOMO energy.
2. **Stable deep states (Groups 1, 2, 5, 6, 7, 8, and 11):** In contrast, most primary amine-based groups and hybrid aromatic amines maintain deeper, more stable HOMO levels (mostly between −5.28 and –5.20 eV, with Groups 6 and 11 slightly lower). This indicates that flexible, unhindered organic layers do not induce a strong electrostatic shift of the perovskite HOMO level.

#### 3.3.2 Lowest Unoccupied Molecular Orbital

The conduction-band edge is most sensitive to the nature of the organic passivation and directly reflects the formation of deep trap states.

1. **Trap-state formation (Groups 4 and 10):** The strongest effect is observed in Groups 4 (pyridinium cations) and 10 (polyaromatics such as pyrene derivatives). In Group 4, the LUMO of the passivated perovskite shifts downward to ≈ −2.70 and −3.13 eV, respectively. This trend is consistent with the low-lying intrinsic LUMO of conjugated organic molecules (e.g., *E*_LUMO_^mol^ ≈ −6.95 eV for Group 4), which can hybridize and lower unoccupied levels at the interface. In Group 10, the stabilization of the LUMO is likely related to reorganization of the electronic structure, possibly mediated by dipole formation.
2. **Confinement (Groups 1, 3, 5, 9, and 12):** Saturated molecules, ranging from small primary amines (Groups 1 and 5) to quaternary ammoniums (Group 3) and sulfoniums (Group 12), maintain a high and stable hybrid LUMO (≈ −0.86 to –0.55 eV). Their wide-gap character and high-energy intrinsic LUMOs act as a dielectric barrier that suppresses electron leakage and trap formation.

#### 3.3.3 Electronic Energy Gap

The electronic gap reflects the combined effects of HOMO and LUMO shifts. The terms “Type I” and “Type II” are formally defined for extended semiconductor heterojunctions; here, we use them as a local, qualitative analogy within the 0D cluster framework to indicate whether adsorption introduces frontier states within the intrinsic gap of the inorganic fragment (trap-like) or leaves that gap essentially unchanged (dielectric/passivating). In this context, a Type I alignment implies that the frontier states of the organic molecule lie outside the pristine perovskite gap, acting as a dielectric barrier. In contrast, a Type II alignment indicates that states with strong organic character intrude into the pristine gap, forming potential trap states.

1. **Gap collapse (Groups 4 and 10):** Due to the lowering of the LUMO, these conjugated groups exhibit collapsed electronic gaps of ≈ 2.24 eV (Group 4) and 1.79 eV (Group 10). This indicates a Type II level alignment, where organic orbitals introduce deep nonradiative recombination states between the core HOMO and LUMO levels.^7^
2. **Gap preservation (Groups 1, 3, 5, 9, 12, and 13):** These groups maintain the wide pristine gap of the perovskite core (≈4.25–4.37 eV). This corresponds to a Type I level alignment, where the organic layer acts as a wide-gap insulator (*E*_g_^mol^ ranging from 7.32 to >8.50 eV) and does not introduce in-gap frontier states in the cluster spectrum.^7^
3. **Intermediate interaction (Groups 2, 6, 7, 8, and 11):** These groups show intermediate hybrid gaps (4.04–4.20 eV), reflecting structural features such as tethered aromatic rings (Group 2) or halogenated tails (Groups 6 and 11) that introduce moderate electronic coupling without a complete gap collapse.

#### 3.3.4 Discussion of Structure–Electronic Relationships

Table 2 synthesizes the physicochemical factors that govern passivation of perovskite fragments. The data show a clear connection between the molecular architecture of the organic adsorbates and the optoelectronic properties at the interface. Two limiting cases can be identified on the basis of steric control and energy-level alignment. Groups 4 and 10, which comprise conjugated resonant species and extended polyaromatics, represent a trap-dominated regime. Their π conjugation stabilizes (lowers) the intrinsic LUMO energy of organic adsorbates, giving rise to a Type II band alignment.^7^ This narrows the effective electronic gap of the perovskite (*E*_g_^mol/PVK^) and promotes nonradiative recombination pathways.

**Table 2:** Interrelationship between Organic Molecular Architecture, Steric Effects, and Electronic Structure Alignment

cluster group	organic chemistry	structural behavior (*d*_donor–Pb_)	electronic mechanism
4 and 10	rigid π-conjugated rings and bulky polyaromatics (e.g., pyrenes, pyridiniums)	intermediate/low: aromaticity dictates specific spatial constraints.	**Type II (Trap):** extremely low intrinsic LUMO strongly couples with the perovskite LUMO, collapsing *E*_g_^mol/PVK^.^7^
3 and 9	highly coordinated, saturated cations (quaternary N, P)	high: massive steric hindrance from bulky central environments.	**Type I (Electrostatic):** wide gap maintained, but surface dipoles push the HOMO upward, slightly destabilizing the HOMO level.^7^
1, 5, and 12	flexible saturated chains and unhindered cations (primary amines, sulfoniums)	low: conformational freedom allows geometric accommodation.	**Type I (Passivator):** wide intrinsic gap creates a dielectric barrier; preserves *E*_g_^mol/PVK^ and a deep HOMO.^7^
2, 6, 7, 8, 11	hybrids (amine + aromatic tether), halogenated chains and resonant amidines	low to Intermediate: Flexible spacers allow close approach.	**Mixed:** moderate electronic coupling or inductive effects slightly reduce *E*_g_^mol/PVK^ without forming deep traps.

By contrast, Groups 1, 5, and 12 correspond to the passivation regime. Comprising conformationally flexible saturated chains and geometrically unhindered headgroups, these molecules exhibit the shortest donor-to-lead distances, allowing efficient structural accommodation at the inorganic interface. From an electronic standpoint, their saturated character yields a large intrinsic electronic gap and enforces a Type I band alignment that acts as a dielectric barrier, preserving the wide electronic gap of the perovskite while maintaining a deep valence-band edge.^7^

Groups 3 and 9 constitute a distinct regime governed primarily by steric crowding and electrostatic effects. The highly coordinated central cations generate pronounced steric hindrance, leading to the largest donor-to-lead separations among the studied systems. Although these molecules retain a wide electronic gap and avoid deep trap formation, their localized positive charge induces an electrostatic upward shift of the valence-band edge (i.e., a higher HOMO level), differentiating this class from the electronically stable passivation behavior observed for the flexible amines. Finally, the intermediate groups (e.g., Group 2) represent hybrid cases where a flexible tether permits close interfacial approach, but functional or aromatic tails introduce moderate electronic coupling, resulting in a modest reduction of the overall electronic gap.

### 3.4 Energetic Properties

To analyze adsorption trends, we separated the energetic contributions into inorganic and organic constituents. The adsorption energy (*E*_ad_) is calculated as follows.^5^

6 $$E_{\mathrm{ad}}=E_{\mathrm{tot}}^{\mathrm{mol}/\mathrm{PVK}}-\left(E_{\mathrm{tot}}^{\mathrm{PVK}}+E_{\mathrm{tot}}^{\mathrm{mol}}\right)$$

where *E*_tot_^mol/*PVK*^, *E*_tot_^PVK^, and *E*_*t*ot_^mol^ are the total energies of the coupled inorganic–organic system, the neutral 0D proxy fragment with three Cs cations, and the neutral, relaxed isolated molecule, respectively. The adsorption energy *E*_ad_ does not separate bonding contributions; therefore, to isolate the electronic interaction at the interface, the interaction energy (*E*_int_) is computed using eq 7.^5^ Here, *E*_tot_^PVK*^ and *E*_tot_^mol*^ are the energies of the fragments frozen in the distorted geometries they adopt in the complex.

7 $$E_{\mathrm{int}}=E_{\mathrm{tot}}^{\mathrm{mol}/\mathrm{PVK}}-\left(E_{\mathrm{tot}}^{\mathrm{PV}K^{*}}+E_{\mathrm{tot}}^{\mathrm{mo}l^{*}}\right)$$

The deformation energy *E*_def_ links *E*_ad_ and *E*_int_, capturing the energetic cost (or gain) of structural rearrangement during adsorption, such that *E*_ad_ = *E*_int_ + *E*_def_. Additionally, charge redistribution is quantified by Δ*Q* via Hirshfeld analysis, defined as the difference between the molecule’s charge in the inorganic–organic system and that of the isolated molecule.

8 $$\Delta Q=Q_{\mathrm{tot}}^{\mathrm{mol}*}-Q_{\mathrm{tot}}^{\mathrm{mol}}$$

#### 3.4.1 Electron Affinity as A Sole Predictor

We initially hypothesized that organic cations with higher gas-phase *EA* would bind more strongly to the electron-rich perovskite surface, but Figure 6 does not support this correlation. For example, Group 4 has the highest electron affinity (*EA* ≈ 4.83 eV) but exhibits one of the least favorable adsorption energies in the data set (*E*_*ad*_ ≈ −3.11 eV). Passivation in 0D fragments is therefore not dictated solely by frontier-orbital alignment. Instead, the most stable systems also minimize positive deformation energies. The energetic picture is hierarchical: electronic attraction (*E*_*int*_) provides the initial driving force, but structural rigidity acts as a filter. Molecules that require large mechanical energy to adapt to the surface (high *E*_def_) do not adsorb strongly, regardless of their electron affinity.

**Figure 6:**
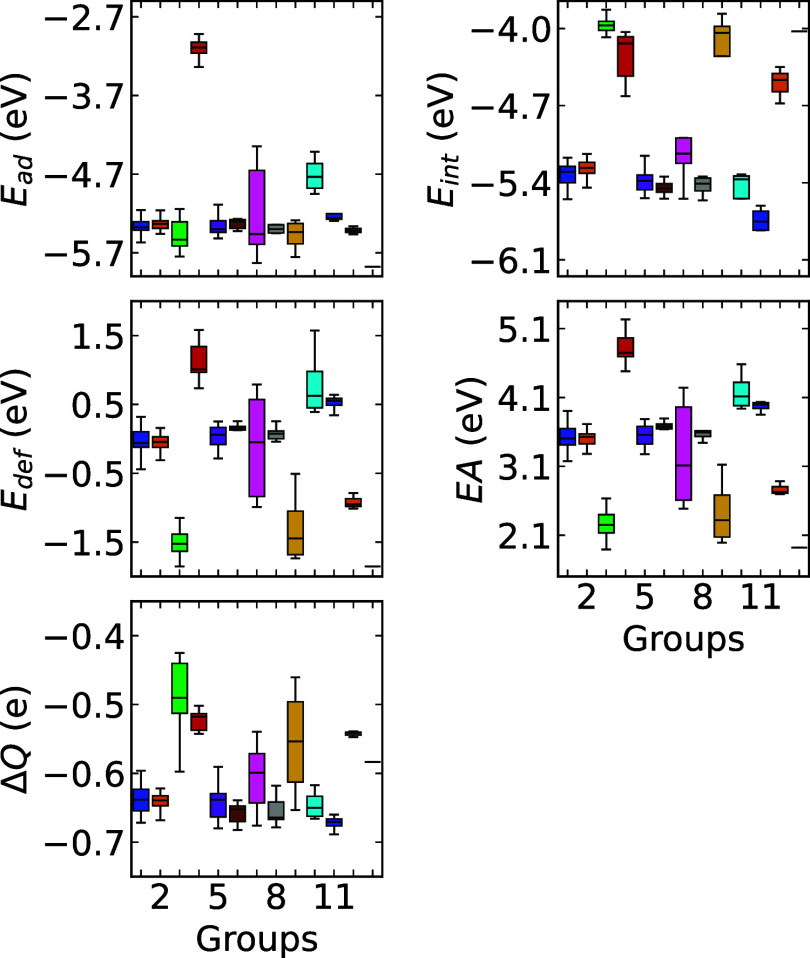
Comparative distribution of energetic and electronic descriptors across the 13 groups. Energetic properties, adsorption energy (*E*_ad_), interaction energy (*E*_int_), molecular electron affinity (*EA*), and deformation energy (*E*_def_), are in eV and obtained from DFT calculations with the HSE06 hybrid functional. Charge transfer, Δ*Q*, in *e*, is computed via Hirshfeld charges using the PBE GGA functional.

#### 3.4.2 Penalty of Structural Rigidity (Groups 4, 7 and 10)

In conjugated systems, such as rigid pyridiniums (Group 4), resonant amidiniums (Group 7), and bulky polyaromatics such as pyrenes (Group 10), steric penalties play a dominant role. Despite favorable electronic properties and moderate-to-strong interaction energies, these groups exhibit the highest structural accommodation penalties or significant deformation variability. For example, Groups 4 and 10 show high average deformation energies (*E*_def_ ≈ +1.12 and ≈+0.80 eV, respectively), while Group 7 exhibits variable behavior centered around *E*_def_ ≈ −0.10 eV. This reflects the resistance of rigid aromatic backbones to conformational changes needed to optimize the interface geometry. As a result, a substantial portion of the interaction energy is spent on mechanical distortion, making net adsorption less predictable and often less favorable than that of flexible chains.

#### 3.4.3 Van der Waals Interactions and Hydrophobic Shielding (Groups 3, 9, and 13)

Coordination-saturated donors (quaternary ammoniums and phosphoniums) show large donor–lead distances due to steric hindrance from surrounding alkyl groups, which weakens electrostatic interactions (*E*_int_ ≈ −4.19–3.97 eV). Nevertheless, they remain stable overall (*E*_ad_ ≈ −5.88-–5.46 eV). Strongly negative deformation energies (*E*_def_ ≈ −1.85–1.28 eV for Group 13) indicate that structural rearrangement during adsorption is driven by intramolecular relaxation and long-range van der Waals interactions. Captured explicitly by the D3 dispersion correction, bulky hydrophobic tails wrap around the inorganic octahedron. This dispersion-driven hydrophobic encapsulation compensates for limited orbital overlap at the binding site.

#### 3.4.4 Strain-Free Electrostatic Anchoring (Groups 1, 2, 5, 6, 8, 11, and 12)

Primary amines and geometrically unhindered sulfoniums define a low-strain passivation regime. Unlike rigid aromatics or sterically hindered quaternary cations, flexible primary and halogenated amines bind with minimal structural penalties. In these families, hydrogen-bond donors N–H (and, in some cases, multiple donor sites) can reinforce interfacial electrostatic attraction through directional hydrogen bonding to surface halides, strengthening anchoring without requiring major backbone distortion. Groups 1, 2, 5, and 8 have deformation energies close to zero (e.g., *E*_def_ ≈ −0.03 eV for Group 1 and 0.03 eV for Group 5), indicating that their molecular geometry matches steric requirements at the perovskite surface. Groups 6 and 11 show small positive values of *E*_def_, reflecting a slight steric mismatch, while sulfoniums (Group 12) show negative *E*_def_, indicating favorable van der Waals accommodation similar to that of coordination-saturated cations. With negligible distortion energy, electrostatic and hydrogen-bonding interactions contribute directly to energetic stability, yielding strong and consistent adsorption energies. This strain-free anchoring indicates that short-to-medium flexible chains are efficient and predictable surface passivators.

## 4 Conclusions

In this study, we combined high-throughput DFT calculations (semilocal and hybrid functionals) with clustering analysis to examine surface passivation in hybrid perovskite fragments. We evaluated the adsorption of 134 post-treatment organic cations on zero-dimensional Cs_4_[PbI_6_] clusters to identify structure–property trends. Comparison with periodic slab calculations (Supporting Information) indicates that the 0D model captures adsorption-energy trends and is suitable for comparative screening. This workflow does not address device-scale processability, and spin–orbit coupling was not included; however, the analysis focuses on relative trends within a consistent computational setup. Purely topological descriptors (e.g., many-body tensor representation) showed limited discriminative power, while physicochemical descriptors separated the data set into 13 groups in which energetic and electronic contributions compete. Overall, passivation is not controlled by a single molecular descriptor (e.g., electron affinity), but by the balance between interfacial interaction and deformation of the organic backbone upon adsorption.

Structurally and energetically, the results show a trade-off between molecular rigidity, steric hindrance, binding energy, and structural deformation. Highly conjugated, rigid systems pay a large energetic penalty (e.g., deformation of ≈1.12 eV), as their limited flexibility channels part of the interaction energy into mechanical distortion and weakens net adsorption. In contrast, bulky, coordination-saturated cations offset weaker electrostatics through London dispersion: their hydrophobic tails wrap around the inorganic core, giving strongly favorable deformation energies (down to *E*_def_ ≈ −1.85 eV) and highlighting the role of van der Waals forces in hydrophobic encapsulation. Flexible primary amines show strain-free electrostatic anchoring: their conformational flexibility enables favorable geometries with negligible deformation cost (*E*_def_ ≈ 0 eV), allowing electrostatics and hydrogen bonding to contribute directly to energetic stability. Similarly, unhindered sulfonium cations gain stability from favorable dispersion, as indicated by their negative deformation energies.

Electronically, the analysis shows a contrast driven by molecular aromaticity and charge localization. Highly π-conjugated molecules (Groups 4 and 10) place low-lying frontier orbitals inside the perovskite gap (Type II alignment), reducing the effective gap (*E*_*g*_^mol/PVK^) and creating deep trap states that promote nonradiative recombination. In contrast, bulky saturated cations with localized positive charge (Groups 3 and 9) shift the HOMO upward via electrostatics, slightly destabilizing the surface. Favorable behavior is observed for saturated, unhindered backbones (Groups 1, 5, and 12), which act as wide-gap insulators, form Type I alignment, and preserve the optoelectronic structure of the perovskite.

These trends indicate that molecular geometry alone is insufficient to capture passivation behavior. We therefore propose the following selection criterion for passivation agents: candidate molecules should combine conformational flexibility (to minimize mechanical penalties and enable strain-free binding) with electronic saturation (to ensure a wide intrinsic HOMO–LUMO separation and avoid Type II trap formation). This criterion rationalizes the empirical success of simple alkylammonium and unhindered sulfonium salts and can inform selection of interface modifiers for perovskite optoelectronic devices.

## Supplementary Material



## Data Availability

The authors declare no competing financial interest. All DFT calculations were performed using the Fritz–Haber Institute *ab initio* molecular simulation package, available under a nonfree academic license.^12^ More details are available in the Supporting Information files. Additional raw data are available from the authors upon request.

## Notes

The data employed for the figures, as well as complementary analyses and additional technical details (ZIP)

## Abbreviations

DFT: density functional theory
PBE: Perdew–Burke–Ernzerhof
FHI-aims: Fritz–Haber Institute *ab initio* molecular simulation
NAOs: numeric atom-centered orbitals
vdW: van der Waals
0D: zero-dimensional
