# Supplementary material for: Combining DFT Calculations and Clustering Techniques to Screen Organic Monovalent Cations for Applications in Halide Perovskite Solar Cells
Source: ACS Omega. 2026 Jun 5;11(24):36169–80. doi: 10.1021/acsomega.6c03327 (PMC13294958; doi:10.1021/acsomega.6c03327)
Supplement: Supplementary file 1 [file ao6c03327_si_001.zip › si_gabrielbueno_israelribeiro_juarez_ODPVK/si1_gabrielbueno_israelribeiro_juarez_ODPVK.pdf]

Electronic Supporting Information: Combining DFT Calculations and Clustering Techniques to Screen Organic Monovalent Cations for Applications in Halide Perovskite Solar Cells

Gabriel C. Bueno,† Israel C. Ribeiro,† Iván Ornelas-Cruz,† Ronaldo C. Prati,‡ Matheus P. Lima,¶ and Juarez L. F. Da Silva*,†

†*São Carlos Institute of Chemistry, University of São Paulo, Av. Trabalhador São-Carlense* 400, 13560-970, *São Carlos, SP, Brazil*

‡*Center of Mathematics, Computation and Cognition, Federal University of ABC, Av. dos Estados,* 5001, 09210-580, *Santo André, SP, Brazil*

¶*Department of Physics, Federal University of São Carlos,* 13565-905, *São Carlos, São Paulo, Brazil*

E-mail: juarez_dasilva@iqsc.usp.br

Contents

S-1 Electronic Supporting Information: Overview **S-2**

S-2 Model Construction and Validation: $Cs_4[PbI_6]$ Fragment **S-2**

S-3 Computational Details and Descriptor Definitions **S-3**

S-3.1 Vertical Electron Affinity S-3

S-3.2 Adsorption and Interaction Energies S-3

S-3.3 Charge Transfer Analysis S-4

S-3.4 Deformation Energy: Structural Distortion Cost S-4

S-3.5 Initial Adsorption Geometries S-4

S-4 Additional Results and Analyses **S-5**

S-4.1 Correlation Analysis and k-Means Clustering S-5
S-4.2 Hierarchical Clustering Results S-17
S-4.3 Summary Tables S-27

References **S-32**

S-1 ELECTRONIC SUPPORTING INFORMATION: OVERVIEW

This Electronic Supporting Information (ESI) summarizes the computational workflow, model construction, and additional analyses supporting our high-throughput DFT screening of 134 organic monovalent cations for halide-perovskite surface passivation.

S-2 MODEL CONSTRUCTION AND VALIDATION: $CS_4[PBI_6]$ FRAGMENT

To evaluate passivation across 134 organic cations, we used a zero-dimensional (0D) $Cs_4[PbI_6]$ cluster as a proxy for an undercoordinated Pb surface site. The 0D fragment focuses on local ligand–Pb interactions and allows the calculation of the energies of adsorption (E_{ad}), interaction (E_{int}), and deformation (E_{def}) within DFT at lower cost than periodic slab models.

To assess the 0D $Cs_4[PbI_6]$ fragment, we benchmark it against 2D periodic slab models. Figure S-1 compares E_{ad} and E_{int} for 16 organic molecules computed with the 0D fragment to values from one- and two-layer 2D models reported by Ribeiro *et al.*[1] The heat map shows agreement and preserves the ranking and trends of the adsorbates. We therefore use the 0D proxy for the large-scale screening reported here.

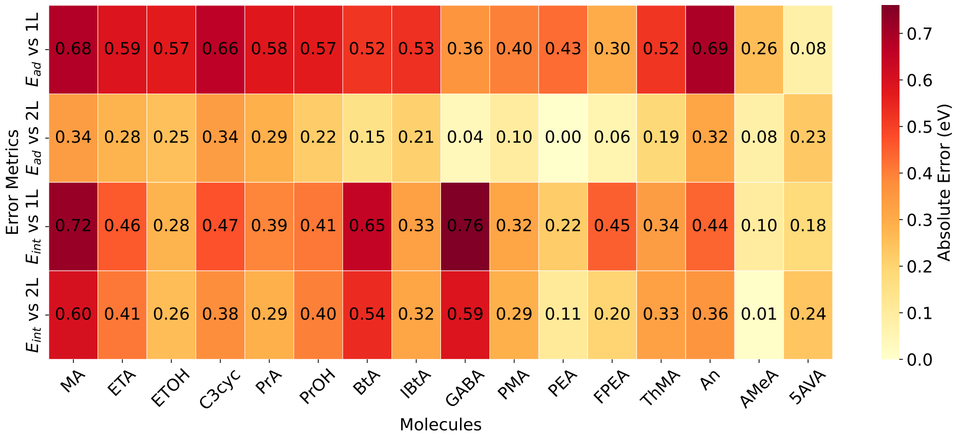

Figure S-1. Heatmap of absolute errors between interaction and adsorption energies computed in this work and literature values for 16 selected systems, previously characterized with periodic one- and two-layer 2D slab models.[1]

S-3 COMPUTATIONAL DETAILS AND DESCRIPTOR DEFINITIONS

S-3.1 Vertical Electron Affinity

We use the vertical electron affinity (EA) as an electronic descriptor of organic cations. EA is defined as the energy gained by adding one electron to the optimized cationic geometry (vertical electron attachment), so larger positive values indicate a stronger tendency to accept the electron density without structural relaxation.

$$EA = E_{tot}^{\text{mol.charged}} - E_{tot}^{\text{mol.neutral (single-point)}} \,. \tag{S-1}$$

Here, $E_{tot}^{\text{mol.charged}}$ is the total energy of the optimized organic cation (net charge +1), and $E_{tot}^{\text{mol.neutral (single-point)}}$ is the total energy of the corresponding neutral molecule evaluated in the cationic geometry (calculation of single-points). We use the vertical definition because some neutral species fragment during geometry optimization, which prevents a consistent evaluation of adiabatic electron affinities.

S-3.2 Adsorption and Interaction Energies

To quantify passivation energetics, we compute two complementary descriptors: the adsorption energy (E_{ad}) and the interaction energy (E_{int}).[1] The adsorption energy measures the thermodynamic stabilization of the neutral passivated complex relative to the separated neutral fragments (0D perovskite proxy + neutral molecule) and is defined as

$$E_{ad} = E_{tot}^{\text{passivated pvk}} - (E_{tot}^{\text{alone pvk}} + E_{tot}^{\text{alone mol}}) \,. \tag{S-2}$$

where $E_{tot}^{\text{passivated pvk}}$ is the total energy of the optimized passivated system, $E_{tot}^{\text{alone pvk}}$ is the total energy of the isolated neutral 0D fragment with three Cs cations (single-point), and $E_{tot}^{\text{alone mol}}$ is the total energy of the isolated neutral molecule (single-point). With this convention, negative E_{ad} values correspond to exothermic (thermodynamically favorable) adsorption.

The interaction energy isolates the electrostatic and electronic interaction between the oppositely charged fragments (organic cation and anionic 0D fragment). It therefore provides

a more direct measure of binding strength that is less sensitive to the reference states of the neutral fragments. It is computed as

$$E_{int} = E_{tot}^{\text{passivated pvk}} - (E_{tot}^{\text{charged alone pvk}} + E_{tot}^{\text{charged alone mol}}) \; . \tag{S-3}$$

where $E_{tot}^{\text{charged alone pvk}}$ is the energy of the isolated 0D fragment with three Cs cations at net charge −1 (single-point), and $E_{tot}^{\text{charged alone mol}}$ is the energy of the isolated organic molecule at net charge +1 (single-point).[1]

S-3.3 Charge Transfer Analysis

To quantify the charge redistribution upon complex formation, we evaluate the net charge transfer (ΔQ) using Hirshfeld partial charges.[2] Specifically, we sum the Hirshfeld charges over all atoms associated with the organic species in the passivated complex and subtract the corresponding sum for the isolated organic cation:

$$\Delta Q = Q_{total}^{mol.pvk} - Q_{total}^{mol} \; . \tag{S-4}$$

With this convention, $\Delta Q < 0$ indicates net electron density transfer from the anionic perovskite fragment to the organic cation (i.e., partial neutralization of the cation upon binding).

S-3.4 Deformation Energy: Structural Distortion Cost

The adsorption energy can be decomposed into an interaction term and a deformation (strain) contribution. The deformation energy, obtained from the difference between the adsorption and interaction energies, represents the energetic penalty associated with structural distortions and the reorganization of the charge-state required to form the bound complex[1].

$$E_{ad} = E_{int} + E_{def} \; . \tag{S-5}$$

S-3.5 Initial Adsorption Geometries

The 0D fragments used for adsorption studies are built from a central octahedron with stoichiometry $Cs_4[PbI_6]$ (Figure S-2). This neutral inorganic core is fully geometry-optimized using the same computational settings adopted for the organic molecules, ensuring

methodological consistency. After optimization, one peripheral Cs atom is replaced by an organic molecule. To sample the potential energy surface and locate stable configurations, the donor group of the organic ligand is initially oriented toward the central Pb atom at a distance of 4 Å, matching the original Cs–Pb separation.

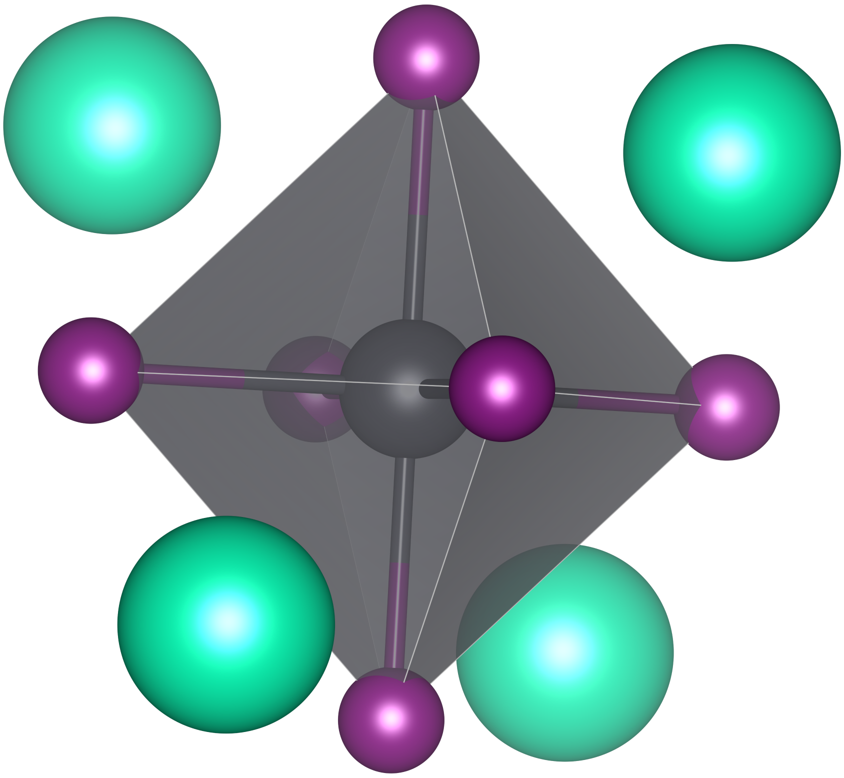

Figure S-2. Structural representation of the zero-dimensional inorganic perovskite fragment $Cs_4[PbI_6]$ in the absence of any organic passivating species.

S-4 ADDITIONAL RESULTS AND ANALYSES

S-4.1 Correlation Analysis and k-Means Clustering

We evaluated pairwise linear correlations among the calculated physicochemical descriptors and tested whether the electron affinity (EA) correlates with the adsorption energy (E_{ad}). We also applied k-means clustering[3] as implemented in `Scikit-learn`[4] using E_{ad}, interaction energy (E_{int}), EA, perovskite band-gap energy ($E_g^{mol/PVK}$), deformation energy (E_{def}), and donor–center distance ($d_{donor-center}$) (Figures S-3–S-13).

The coefficient of determination for EA versus E_{ad} was $R^2 = 0.51$, indicating only a moderate linear correlation. Stronger correlations were found for charge transfer (ΔQ) versus E_{int} and for $d_{donor-center}$ versus E_{int} (both $R^2 = 0.84$). The highest correlation was observed between EA and

E_{def} ($R^2 = 0.98$), which is expected from the definitions employed: as shown in Equation S-6, E_{def} contains the term $(E_{tot}^{\text{charged alone mol}} - E_{tot}^{\text{alone mol}})$, which closely mirrors the definition of EA. Summary statistics are reported in Table S-1.

$$E_{def} = (E_{tot}^{\text{charged alone pvk}} - E_{tot}^{\text{alone pvk}}) + (E_{tot}^{\text{charged alone mol}} - E_{tot}^{\text{alone mol}}) \, . \quad \text{(S-6)}$$

Table S-1. Mean and standard deviation of the terms contributing to the deformation energy E_{def} (Equation S-6).

Property	Energy (eV)
EA	3.344 ± 0.722
$(E_{tot}^{\text{charged alone pvk}} - E_{tot}^{\text{alone pvk}})$	−3.682 ± 0.059
$(E_{tot}^{\text{charged alone mol}} - E_{tot}^{\text{alone mol}})$	3.437 ± 0.754

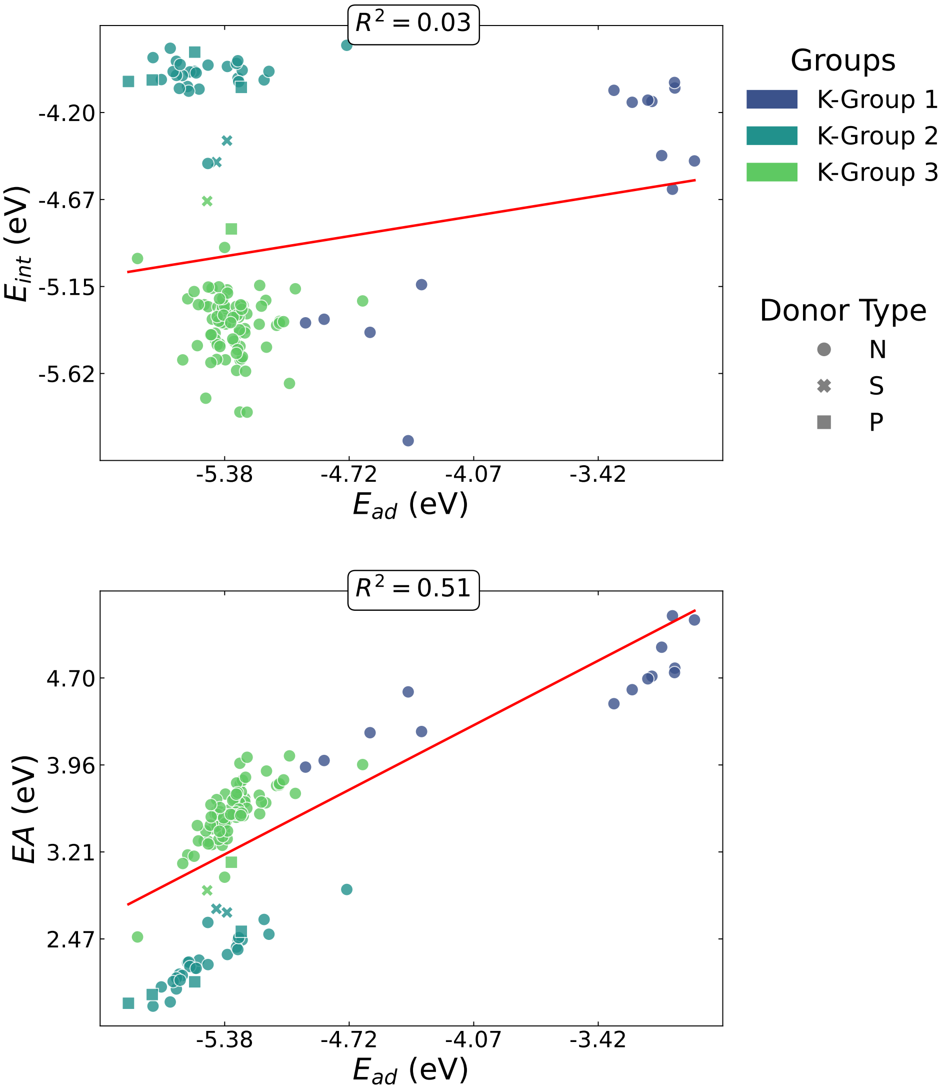

Figure S-3. Correlation plots for physicochemical descriptors, with points colored by *k*-means cluster assignment. Markers indicate the primary donor element: circles (N-based), crosses (S-based), and squares (P-based). Top: adsorption energy (E_{ad}) versus interaction energy (E_{int}) ($R^2 = 0.03$). Bottom: E_{ad} versus electron affinity (EA) ($R^2 = 0.51$). Energies are in eV.

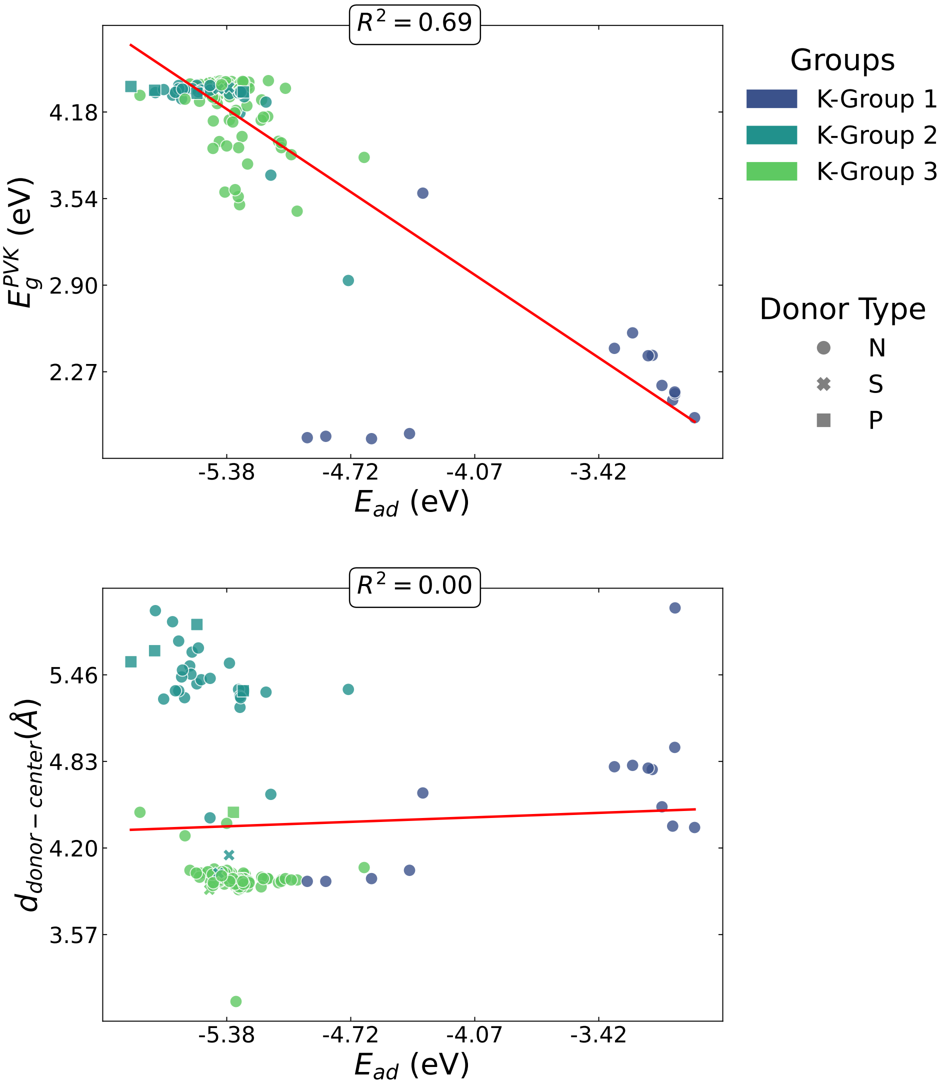

Figure S-4. Correlation plots with points colored by k-means cluster assignment and markers indicating the primary donor element (circles: N-based; crosses: S-based; squares: P-based). Top: E_{ad} versus perovskite band gap ($E_g^{mol/PVK}$) ($R^2 = 0.69$). Bottom: E_{ad} versus donor–metal center distance ($d_{donor-center}$) ($R^2 = 0.00$). Energies are in eV and distances in Å.

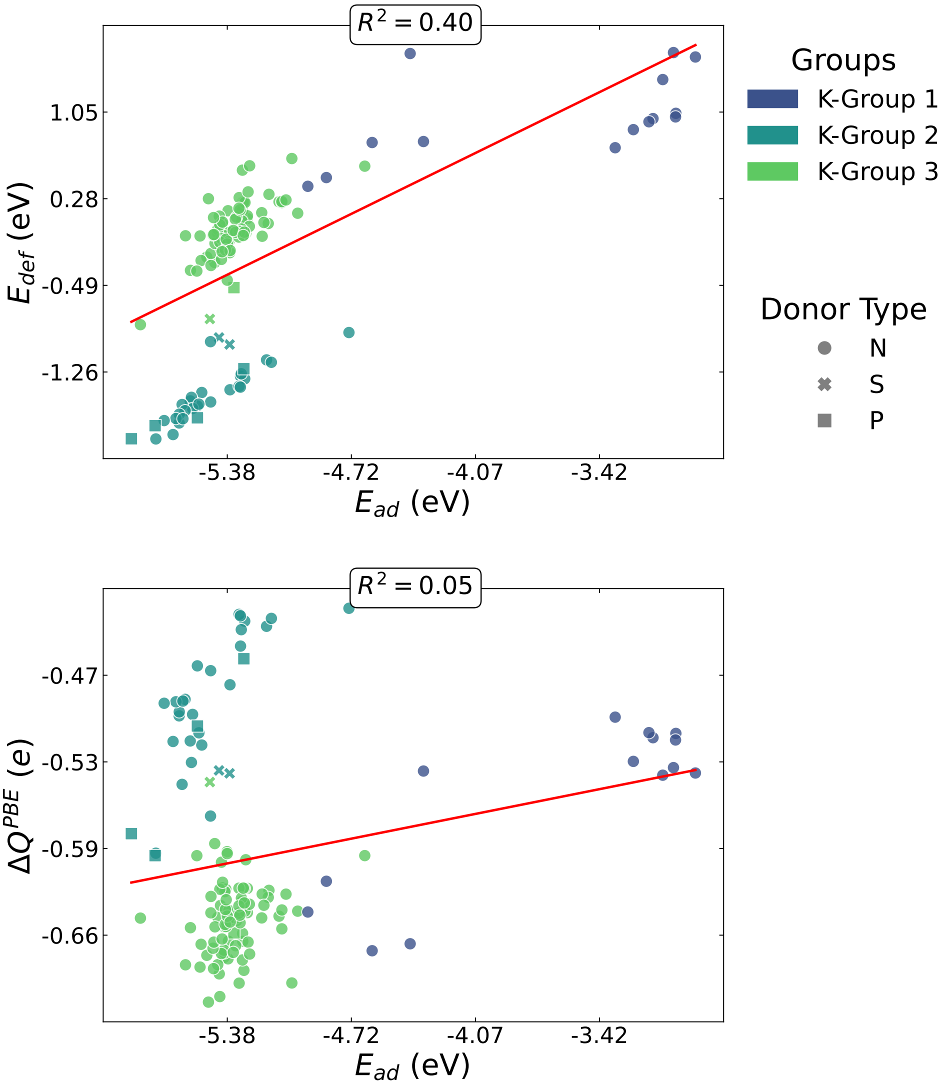

Figure S-5. Correlation plots with points colored by k-means cluster assignment and markers indicating the primary donor element (circles: N-based; crosses: S-based; squares: P-based). Top: E_{ad} versus deformation energy (E_{def}) ($R^2 = 0.40$). Bottom: E_{ad} versus charge transfer (ΔQ) ($R^2 = 0.05$). Energies are in eV.

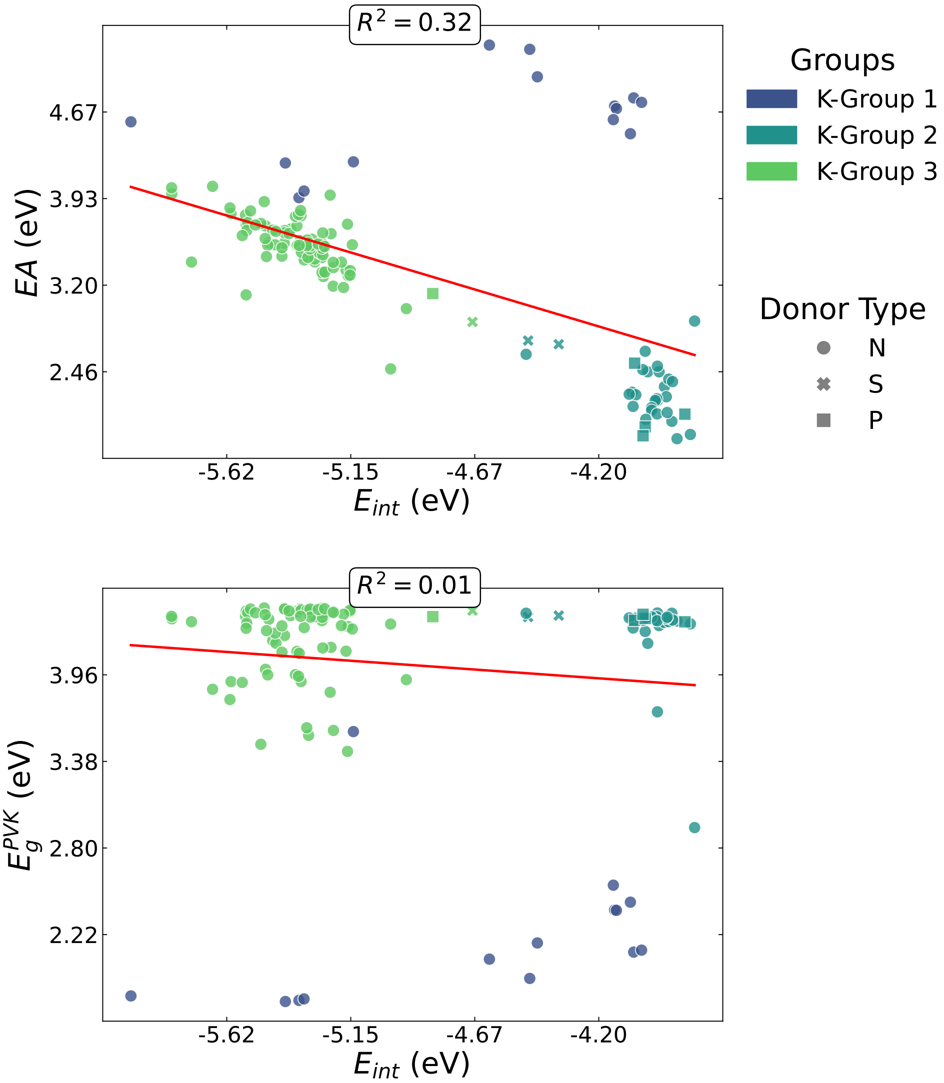

Figure S-6. Correlation plots with points colored by *k*-means cluster assignment and markers indicating the primary donor element (circles: N-based; crosses: S-based; squares: P-based). Top: E_{int} versus EA ($R^2 = 0.32$). Bottom: E_{int} versus perovskite band gap ($E_g^{mol/PVK}$) ($R^2 = 0.01$). Energies are in eV.

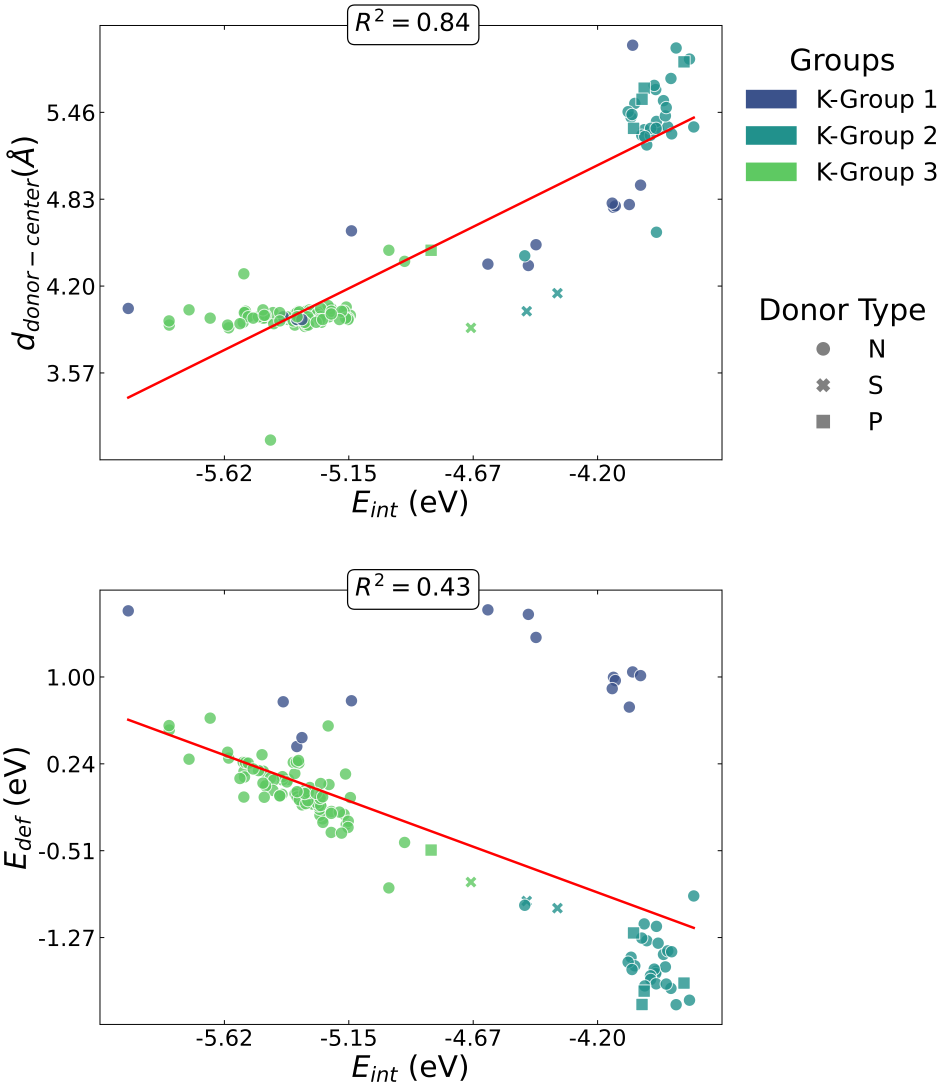

Figure S-7. Correlation plots with points colored by k-means cluster assignment and markers indicating the primary donor element (circles: N-based; crosses: S-based; squares: P-based). Top: E_{int} versus $d_{donor-center}$ ($R^2 = 0.84$). Bottom: E_{int} versus E_{def} ($R^2 = 0.43$). Energies are in eV and distances in Å.

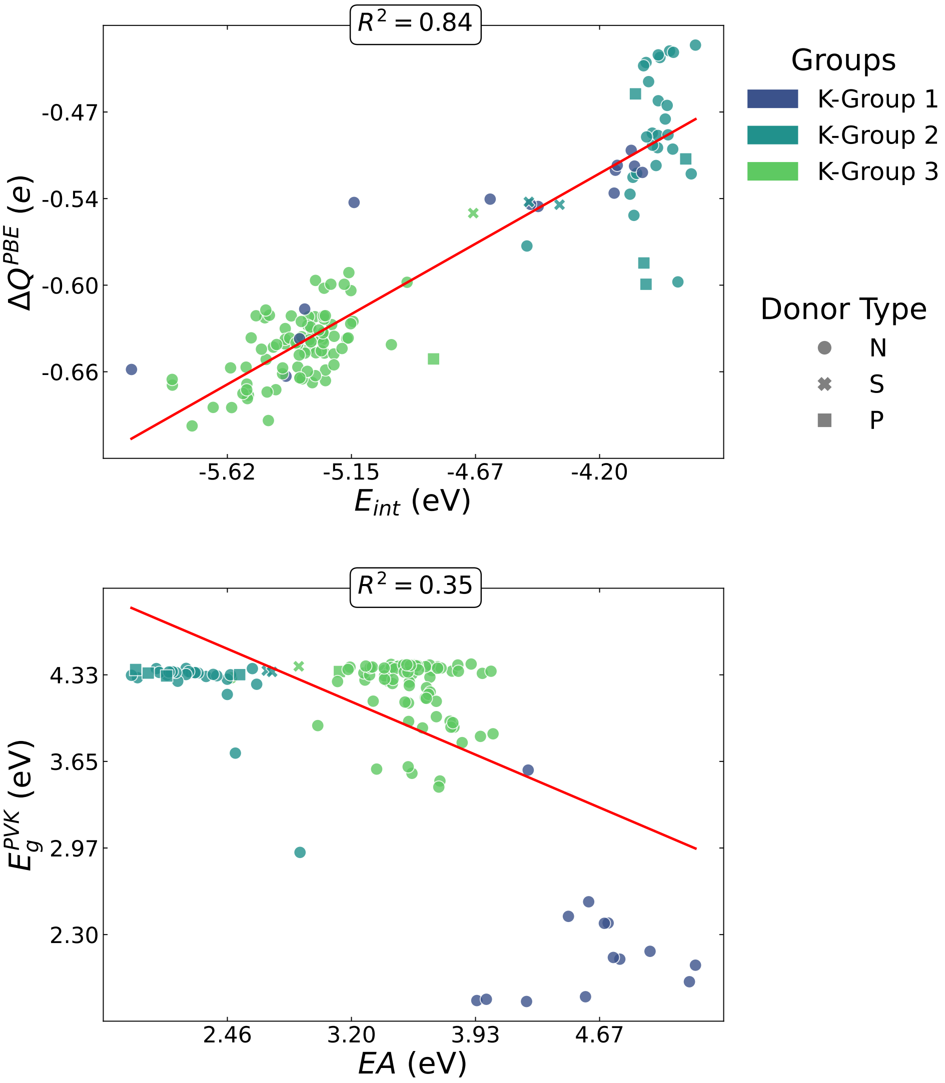

Figure S-8. Correlation plots with points colored by k-means cluster assignment and markers indicating the primary donor element (circles: N-based; crosses: S-based; squares: P-based). Top: E_{int} versus ΔQ ($R^2 = 0.84$). Bottom: EA versus $E_g^{mol/PVK}$ ($R^2 = 0.35$). Energies are in eV.

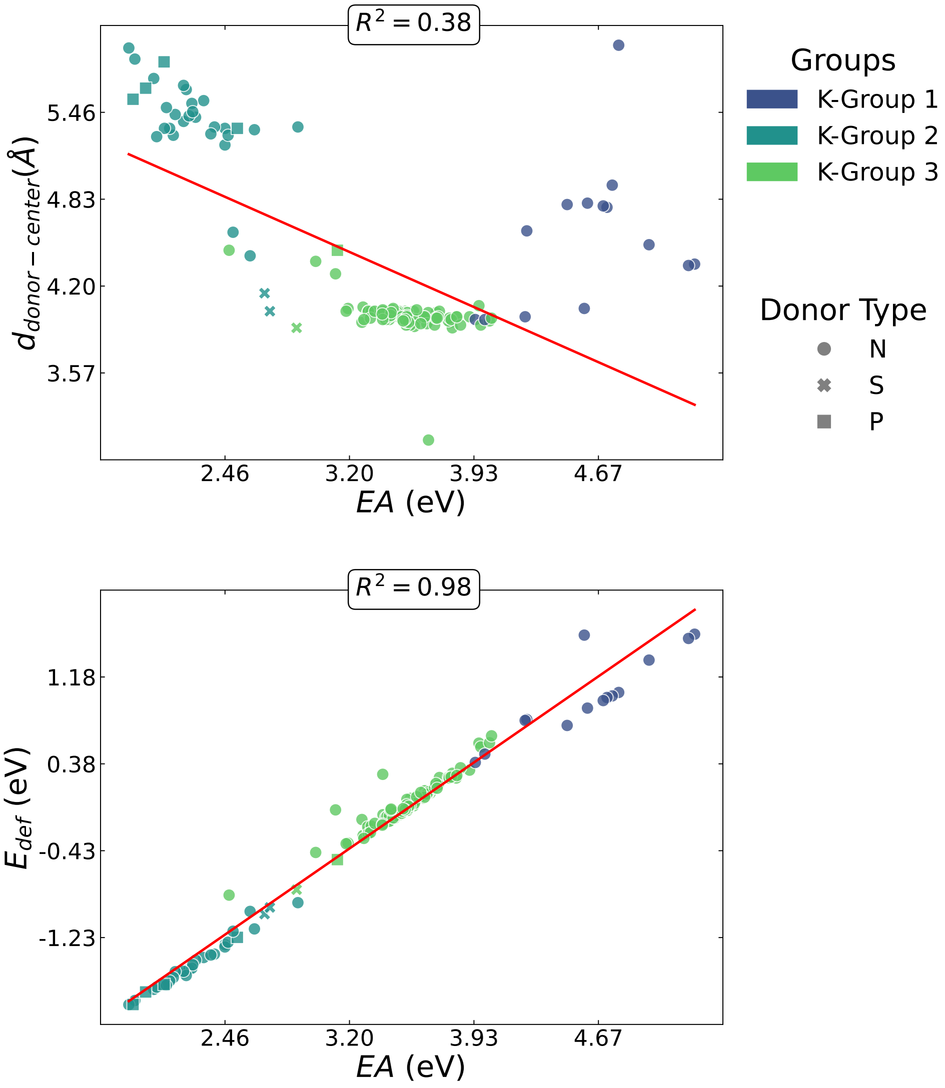

Figure S-9. Correlation plots with points colored by *k*-means cluster assignment and markers indicating the primary donor element (circles: N-based; crosses: S-based; squares: P-based). Top: EA versus $d_{donor-center}$ (R^2 = 0.38). Bottom: EA versus E_{def} (R^2 = 0.98). Energies are in eV and distances in Å.

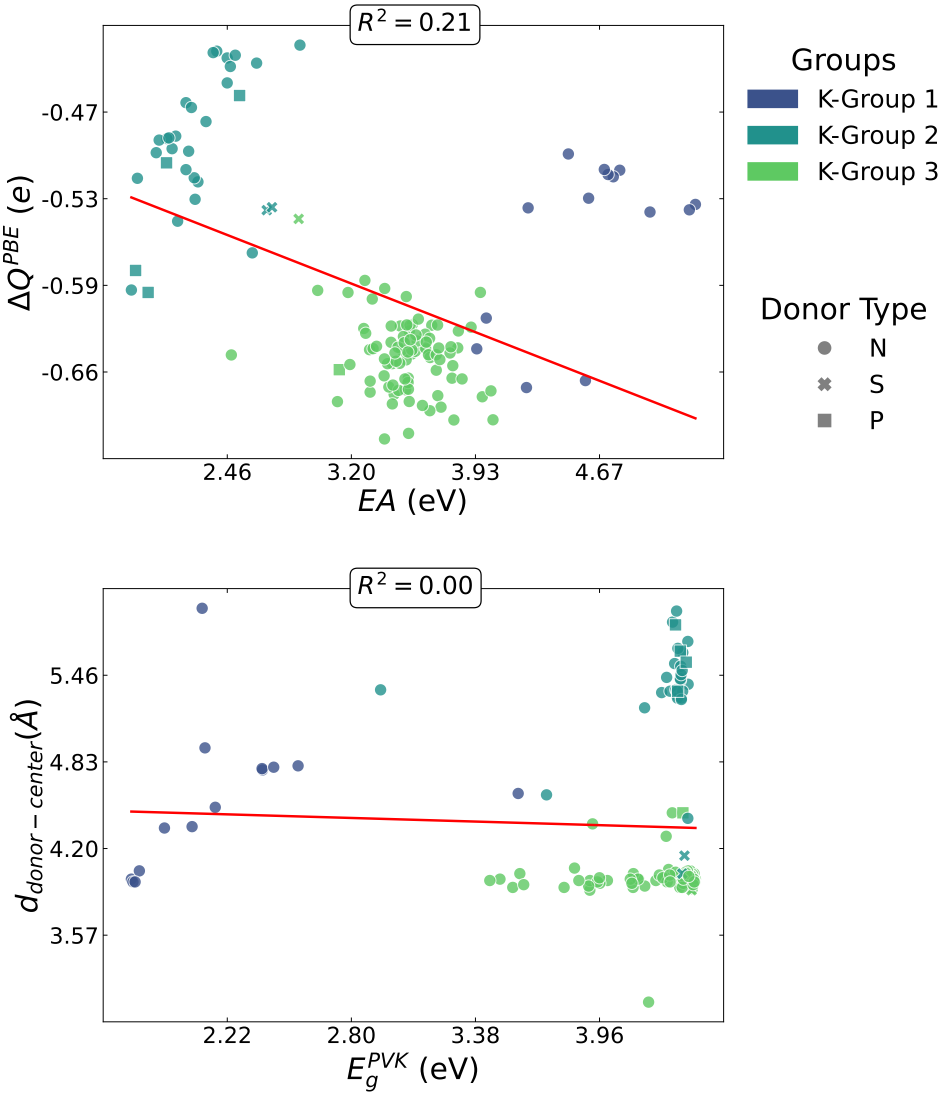

Figure S-10. Correlation plots with points colored by k-means cluster assignment and markers indicating the primary donor element (circles: N-based; crosses: S-based; squares: P-based). Top: EA versus ΔQ ($R^2 = 0.21$). Bottom: $E_g^{mol/PVK}$ versus $d_{donor-center}$ ($R^2 = 0.00$). Energies are in eV and distances in Å.

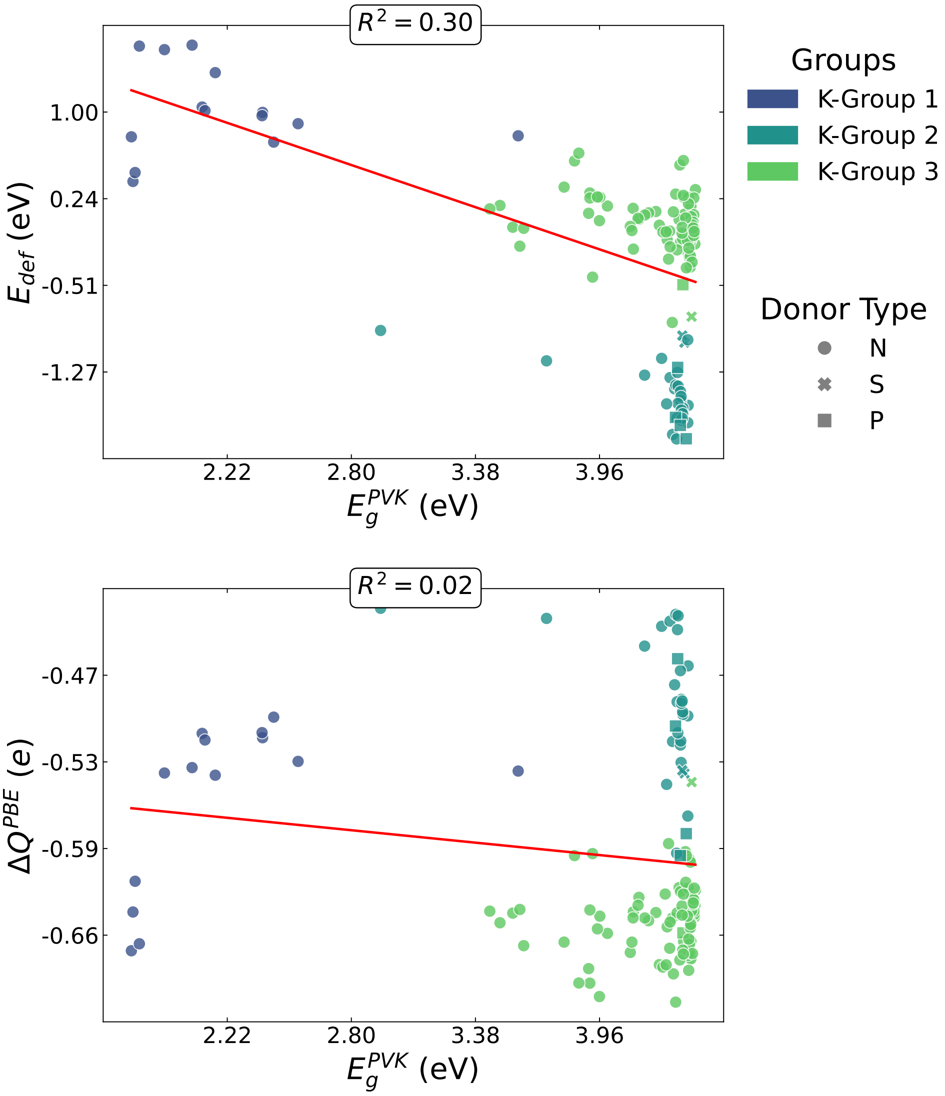

Figure S-11. Correlation plots with points colored by k-means cluster assignment and markers indicating the primary donor element (circles: N-based; crosses: S-based; squares: P-based). Top: $E_g^{mol/PVK}$ versus E_{def} ($R^2 = 0.30$). Bottom: $E_g^{mol/PVK}$ versus ΔQ ($R^2 = 0.02$). Energies are in eV.

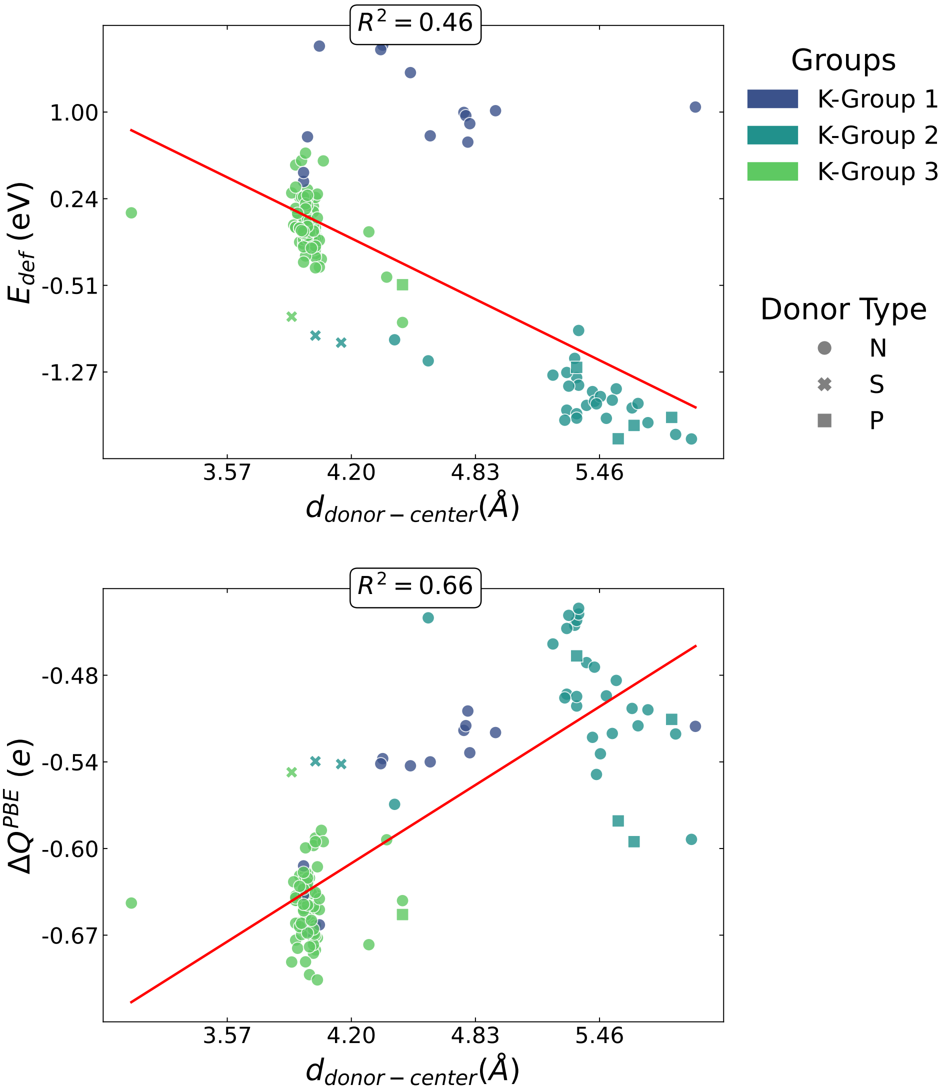

Figure S-12. Correlation plots with points colored by k-means cluster assignment and markers indicating the primary donor element (circles: N-based; crosses: S-based; squares: P-based). Top: $d_{donor-center}$ versus E_{def} (R^2 = 0.46). Bottom: $d_{donor-center}$ versus ΔQ (R^2 = 0.66). Energies are in eV and distances in Å.

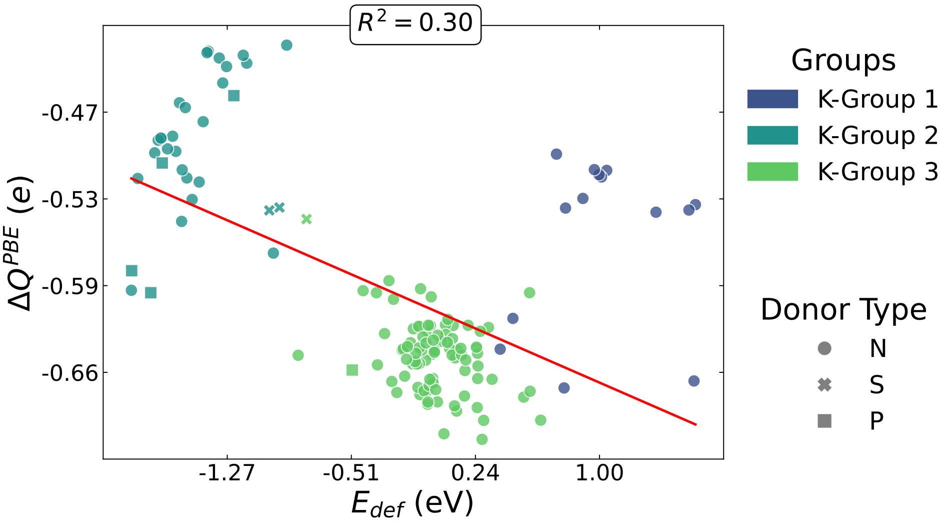

Figure S-13. Correlation plot with points colored by k-means cluster assignment and markers indicating the primary donor element (circles: N-based; crosses: S-based; squares: P-based): E_{def} versus ΔQ (R^2 = 0.30). Energies are in eV.

S-4.2 Hierarchical Clustering Results

We used hierarchical agglomerative clustering to group organic monovalent cations and relate chemistry to computed descriptors.[4] Clustering employed Ward linkage[5] with Euclidean distances to minimize variance within the cluster. Hierarchical clustering was applied independently to three feature spaces: (i) physicochemical descriptor vectors from our DFT calculations (structural, energetic, and electronic properties); (ii) Many-Body Tensor Representation (MBTR) vectors for isolated organic molecules; and (iii) MBTR vectors for fully passivated perovskite complexes. The distance cutoff points of 12 (physicochemical descriptors), 352 (molecular MBTR) and 351 (complex MBTR) yielded G = 13 groups. Figures S-14–S-16 show the corresponding dendrograms, and Figures S-17–S-19 summarize the group-wise distributions.

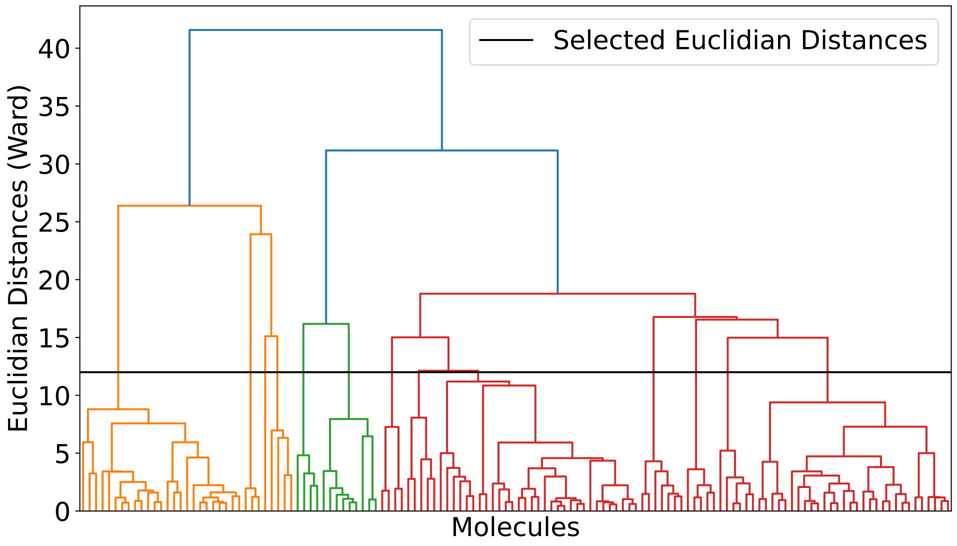

Figure S-14. Hierarchical-clustering dendrogram for the passivated systems using physicochemical descriptor vectors. Clustering employed Ward linkage with Euclidean distances. The solid black horizontal line at distance 12 indicates the cutoff used to partition the dataset into G = 13 functional groups.

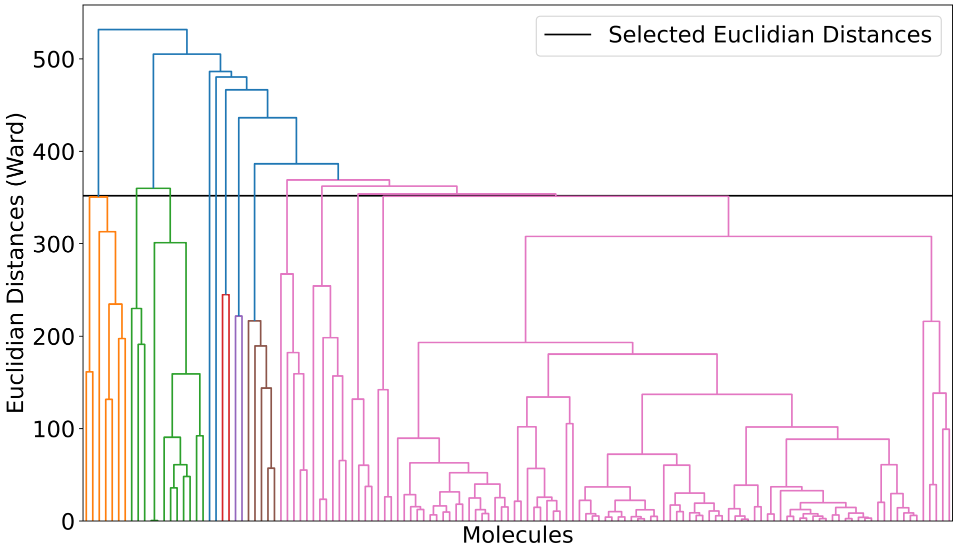

Figure S-15. Hierarchical-clustering dendrogram using MBTR vectors of the isolated organic molecules. Clustering employed Ward linkage with Euclidean distances. The solid black horizontal line at distance 352 indicates the cutoff used to partition the dataset into G = 13 functional groups.

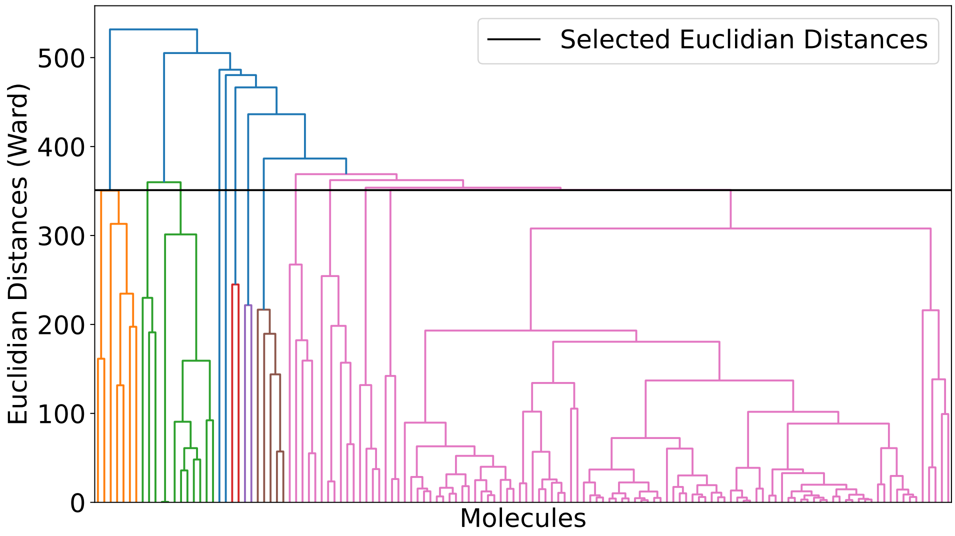

Figure S-16. Hierarchical-clustering dendrogram using MBTR vectors of the fully passivated perovskite complexes. Clustering employed Ward linkage with Euclidean distances. The solid black horizontal line at distance 351 indicates the cutoff used to partition the dataset into $G = 13$ functional groups.

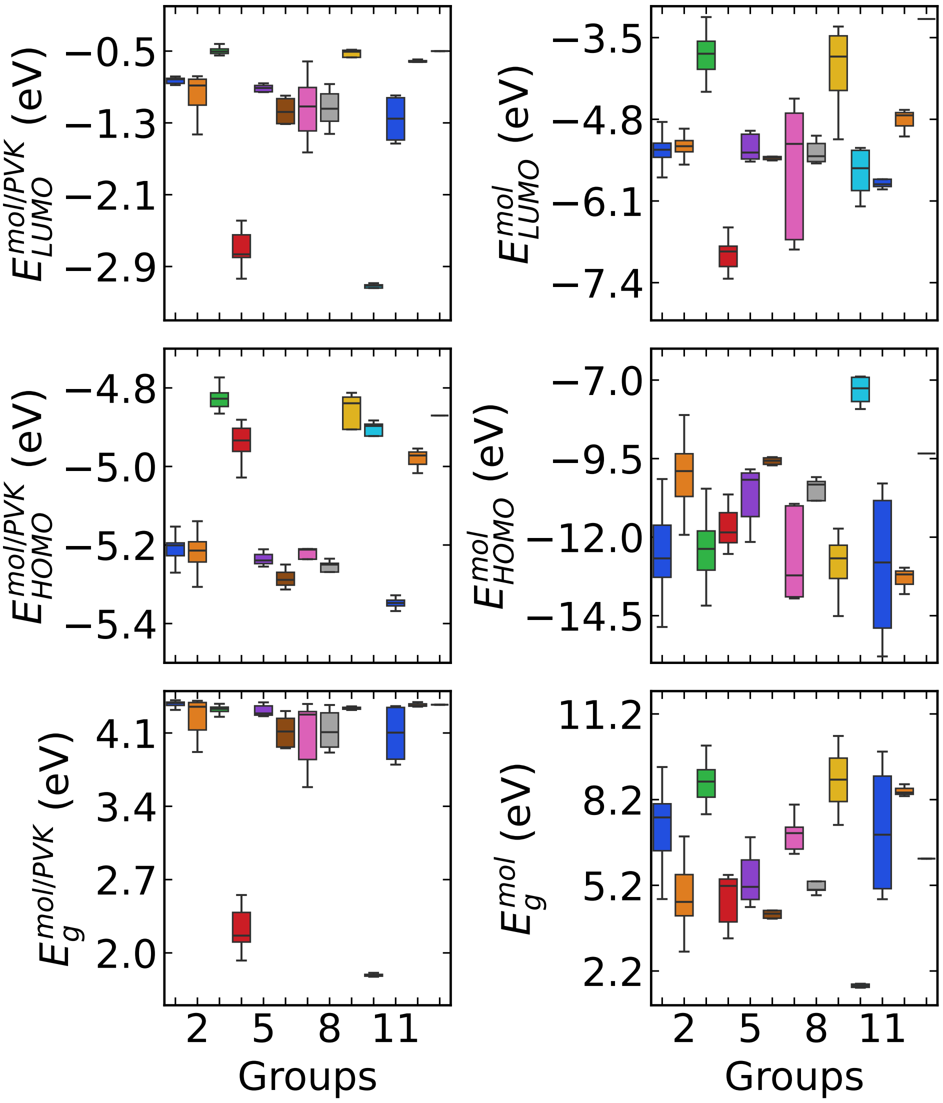

Figure S-17. Distribution of electronic descriptors across the 13 hierarchical-clustering (HC) groups obtained with the HSE06 hybrid functional. Left: passivated perovskites ($E_{\mathrm{LUMO}}^{\mathrm{mol/PVK}}$, $E_{\mathrm{HOMO}}^{\mathrm{mol/PVK}}$, and $E_{g}^{\mathrm{mol/PVK}}$). Right: isolated organic molecules ($E_{\mathrm{LUMO}}^{\mathrm{mol}}$, $E_{\mathrm{HOMO}}^{\mathrm{mol}}$, and E_{g}^{mol}). Energies are in eV.

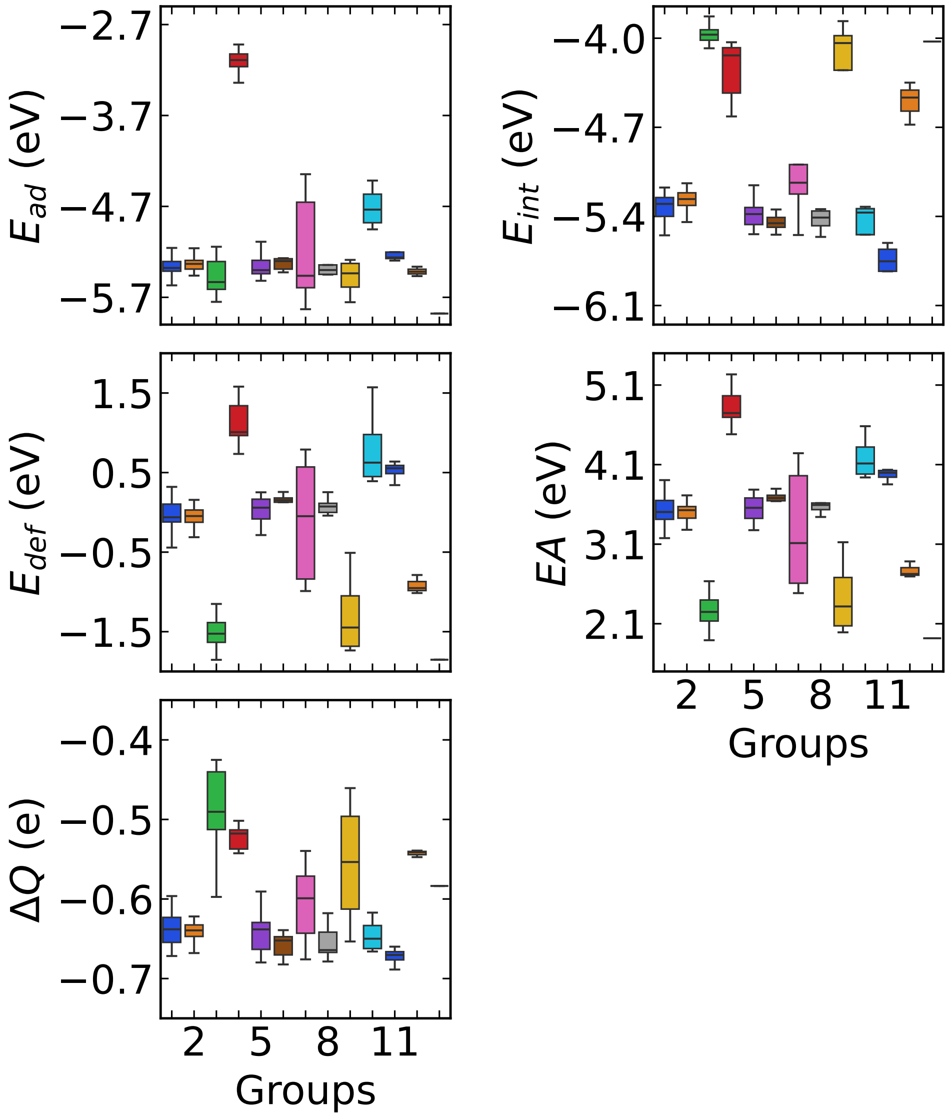

Figure S-18. Distribution of energetic descriptors across the 13 HC groups. E_{ad}, E_{int}, EA, and E_{def} are in eV and were obtained from DFT calculations using HSE06. Charge transfer (ΔQ, in e) was computed from Hirshfeld charges using the PBE GGA functional.

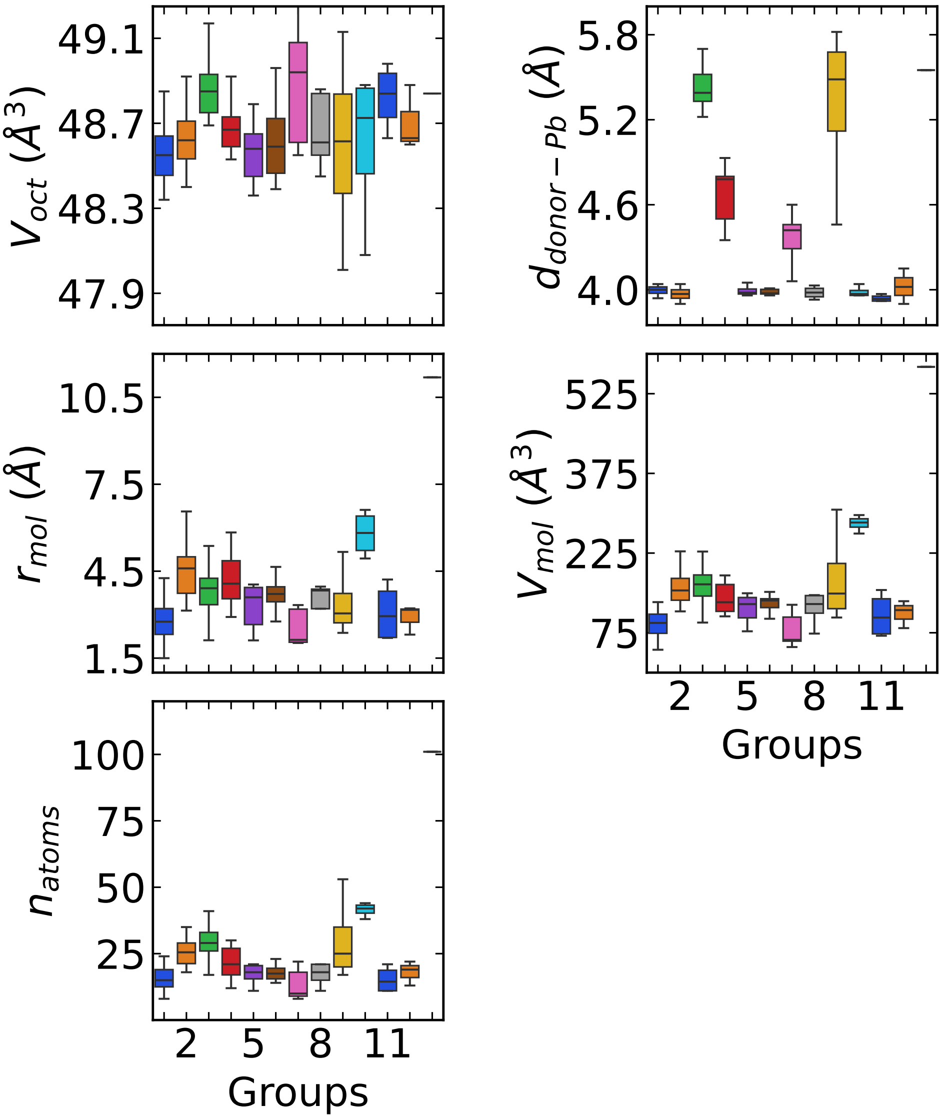

Figure S-19. Distribution of structural descriptors across the 13 HC groups. Points with overlaid boxplots show dispersion and central tendency. Structural parameters include the iodine-octahedra volume (V_{oct}), donor–Pb distance ($d_{\text{donor-Pb}}$), average molecular radius (r_{mol}), molecular volume (V_{mol}), and number of atoms (n_{atoms}). Volumes are in $Å^3$ and distances in Å.

Table S-2. Summary of compositional, structural, electronic, and energetic descriptors (mean ± standard deviation) for Groups 1–3. Energies are in eV, ΔQ in e, volumes in Å^3, and distances in Å.

Property	**Group 1**	**Group 2**	**Group 3**
N %	100.0	100.0	100.0
P %	0.0	0.0	0.0
S %	0.0	0.0	0.0
V_{oct}	48.57 ± 0.16	48.64 ± 0.15	48.81 ± 0.25
$d_{\text{donor-Pb}}$	4.01 ± 0.07	3.94 ± 0.17	5.42 ± 0.25
r_{mol}	2.83 ± 0.64	4.74 ± 1.34	3.84 ± 0.79
V_{mol}	92.45 ± 23.81	165.12 ± 41.83	164.15 ± 41.55
n_{atoms}	16.00 ± 4.19	27.57 ± 8.14	29.28 ± 7.49
$E_{LUMO}^{mol/PVK}$	−0.86 ± 0.10	−1.02 ± 0.30	−0.58 ± 0.29
$E_{HOMO}^{mol/PVK}$	−5.21 ± 0.04	−5.22 ± 0.04	−4.83 ± 0.03
$E_g^{mol/PVK}$	4.35 ± 0.10	4.20 ± 0.29	4.24 ± 0.30
E_{LUMO}^{mol}	−5.29 ± 0.24	−5.22 ± 0.15	−3.79 ± 0.35
E_{HOMO}^{mol}	−12.61 ± 1.42	−9.99 ± 0.95	−12.48 ± 1.13
E_g^{mol}	7.32 ± 1.37	4.78 ± 1.03	8.69 ± 1.20
E_{ad}	−5.37 ± 0.11	−5.33 ± 0.09	−5.46 ± 0.23
E_{int}	−5.34 ± 0.15	−5.28 ± 0.10	−3.97 ± 0.06
E_{def}	−0.03 ± 0.20	−0.05 ± 0.13	−1.49 ± 0.22
EA	3.51 ± 0.20	3.51 ± 0.13	2.27 ± 0.22
ΔQ	−0.64 ± 0.02	−0.64 ± 0.02	−0.48 ± 0.04
Number of Materials	31	30	25

Table S-3. Summary of compositional, structural, electronic, and energetic descriptors (mean ± standard deviation) for Groups 4–6. Energies are in eV, ΔQ in e, volumes in $Å^3$, and distances in Å.

Property	**Group 4**	**Group 5**	**Group 6**
N %	100.0	100.0	100.0
P %	0.0	0.0	0.0
S %	0.0	0.0	0.0
V_{oct}	48.63 ± 0.20	48.51 ± 0.27	48.62 ± 0.21
$d_{\text{donor-Pb}}$	4.80 ± 0.47	3.99 ± 0.03	3.98 ± 0.02
r_{mol}	4.23 ± 0.94	3.28 ± 0.79	3.71 ± 0.63
V_{mol}	138.71 ± 28.07	120.70 ± 27.36	130.46 ± 17.91
n_{atoms}	21.44 ± 5.94	17.43 ± 3.78	17.83 ± 3.31
$E_{LUMO}^{mol/PVK}$	−2.70 ± 0.23	−0.97 ± 0.16	−1.25 ± 0.30
$E_{HOMO}^{mol/PVK}$	−4.94 ± 0.04	−5.23 ± 0.05	−5.29 ± 0.02
$E_{g}^{mol/PVK}$	2.24 ± 0.21	4.27 ± 0.16	4.04 ± 0.30
E_{LUMO}^{mol}	−6.95 ± 0.28	−5.25 ± 0.22	−5.40 ± 0.07
E_{HOMO}^{mol}	−11.64 ± 0.72	−10.68 ± 0.97	−9.55 ± 0.44
E_{g}^{mol}	4.69 ± 0.91	5.44 ± 1.02	4.16 ± 0.43
E_{ad}	−3.11 ± 0.13	−5.35 ± 0.14	−5.30 ± 0.12
E_{int}	−4.23 ± 0.21	−5.38 ± 0.13	−5.45 ± 0.07
E_{def}	1.12 ± 0.29	0.03 ± 0.19	0.15 ± 0.08
EA	4.83 ± 0.26	3.55 ± 0.18	3.68 ± 0.09
ΔQ	−0.52 ± 0.01	−0.64 ± 0.03	−0.66 ± 0.02
Number of Materials	9	7	6

Table S-4. Summary of compositional, structural, electronic, and energetic descriptors (mean ± standard deviation) for Groups 7–9. Energies are in eV, ΔQ in e, volumes in Å^3, and distances in Å.

Property	**Group 7**	**Group 8**	**Group 9**
N %	100.0	100.0	0.0
P %	0.0	0.0	100.0
S %	0.0	0.0	0.0
V_{oct}	48.89 ± 0.31	48.66 ± 0.18	48.59 ± 0.47
$d_{\text{donor-Pb}}$	4.37 ± 0.20	3.98 ± 0.04	5.31 ± 0.60
r_{mol}	2.54 ± 0.66	3.40 ± 0.78	3.41 ± 1.23
V_{mol}	80.24 ± 34.11	121.05 ± 30.02	177.00 ± 90.99
n_{atoms}	13.40 ± 6.23	17.20 ± 4.27	30.00 ± 16.12
$E_{LUMO}^{mol/PVK}$	−1.13 ± 0.40	−1.14 ± 0.22	−0.55 ± 0.11
$E_{HOMO}^{mol/PVK}$	−5.21 ± 0.14	−5.27 ± 0.04	−4.89 ± 0.12
$E_{g}^{mol/PVK}$	4.08 ± 0.35	4.13 ± 0.20	4.34 ± 0.02
E_{LUMO}^{mol}	−5.59 ± 1.13	−5.32 ± 0.19	−4.01 ± 0.80
E_{HOMO}^{mol}	−12.60 ± 1.52	−10.87 ± 1.15	−12.90 ± 1.18
E_{g}^{mol}	7.01 ± 0.68	5.55 ± 1.09	8.89 ± 1.33
E_{ad}	−5.18 ± 0.64	−5.35 ± 0.14	−5.48 ± 0.21
E_{int}	−5.07 ± 0.39	−5.43 ± 0.09	−4.19 ± 0.43
E_{def}	−0.10 ± 0.80	0.08 ± 0.11	−1.29 ± 0.56
EA	3.28 ± 0.79	3.59 ± 0.12	2.44 ± 0.51
ΔQ	−0.61 ± 0.05	−0.65 ± 0.02	−0.56 ± 0.09
Number of Materials	5	5	4

Table S-5. Summary of compositional, structural, electronic, and energetic descriptors (mean ± standard deviation) for Groups 10–12. Energies are in eV, ΔQ in e, volumes in $Å^3$, and distances in Å.

Property	**Group 10**	**Group 11**	**Group 12**
N %	100.0	100.0	0.0
P %	0.0	0.0	0.0
S %	0.0	0.0	100.0
V_{oct}	48.60 ± 0.37	48.82 ± 0.16	48.70 ± 0.15
$d_{\text{donor-Pb}}$	3.99 ± 0.04	3.94 ± 0.02	4.02 ± 0.13
r_{mol}	5.80 ± 0.80	3.08 ± 1.03	2.90 ± 0.51
V_{mol}	280.83 ± 14.84	108.10 ± 42.98	111.92 ± 25.82
n_{atoms}	41.50 ± 2.65	15.25 ± 5.06	18.00 ± 4.58
$E_{LUMO}^{mol/PVK}$	−3.13 ± 0.05	−1.26 ± 0.29	−0.61 ± 0.02
$E_{HOMO}^{mol/PVK}$	−4.92 ± 0.05	−5.35 ± 0.02	−4.98 ± 0.03
$E_{g}^{mol/PVK}$	1.79 ± 0.02	4.09 ± 0.30	4.37 ± 0.02
E_{LUMO}^{mol}	−5.65 ± 0.45	−5.79 ± 0.15	−4.82 ± 0.22
E_{HOMO}^{mol}	−7.33 ± 0.51	−12.92 ± 2.68	−13.32 ± 0.44
E_{g}^{mol}	1.68 ± 0.07	7.13 ± 2.56	8.50 ± 0.21
E_{ad}	−4.71 ± 0.24	−5.21 ± 0.12	−5.42 ± 0.05
E_{int}	−5.51 ± 0.32	−5.74 ± 0.11	−4.50 ± 0.17
E_{def}	0.80 ± 0.54	0.52 ± 0.13	−0.92 ± 0.12
EA	4.19 ± 0.29	3.97 ± 0.08	2.77 ± 0.10
ΔQ	−0.65 ± 0.02	−0.67 ± 0.01	−0.54 ± 0.00
Number of Materials	4	4	3

Table S-6. Summary of compositional, structural, electronic, and energetic descriptors (mean ± standard deviation) for Group 13. Energies are in eV, ΔQ in e, volumes in Å^3, and distances in Å.

Property	**Group 13**
N %	0.0
P %	100.0
S %	0.0
V_{oct}	48.84 ± 0.00
$d_{\text{donor-Pb}}$	5.55 ± 0.00
r_{mol}	11.19 ± 0.00
V_{mol}	575.52 ± 0.00
n_{atoms}	101.00 ± 0.00
$E_{LUMO}^{mol/PVK}$	−0.50 ± 0.00
$E_{HOMO}^{mol/PVK}$	−4.87 ± 0.00
$E_g^{mol/PVK}$	4.37 ± 0.00
E_{LUMO}^{mol}	−3.20 ± 0.00
E_{HOMO}^{mol}	−9.34 ± 0.00
E_g^{mol}	6.13 ± 0.00
E_{ad}	−5.88 ± 0.00
E_{int}	−4.03 ± 0.00
E_{def}	−1.85 ± 0.00
EA	1.92 ± 0.00
ΔQ	−0.58 ± 0.00
Number of Materials	1

S-4.3 Summary Tables

Table S-7. Molecules assigned to Group 1 and their energetic/electronic descriptors: interaction energy (E_{int}), adsorption energy (E_{ad}), deformation energy (E_{def}), perovskite band gap ($E_g^{mol/PVK}$), and molecular band gap (E_g^{mol}). Energies are in eV.

Molecule	E_{int}	E_{ad}	E_{def}	$E_g^{mol/PVK}$	E_g^{mol}
Methylammonium	−5.48	−5.16	0.32	4.41	11.08
Ethylammonium	−5.38	−5.27	0.11	4.36	9.34
2-aminoethanol protonated	−5.28	−5.38	−0.09	4.37	7.58
Cyclopropylammonium	−5.40	−5.42	−0.02	4.41	8.08
Propylammonium	−5.34	−5.35	−0.01	4.41	8.49
But-3-yn-1-ammonium	−5.55	−5.29	0.25	4.36	5.61
Cyclobutylammonium	−5.29	−5.38	−0.10	4.40	7.98
3-aminopropanol protonated	−5.25	−5.28	−0.03	4.39	6.04
Isobutylammonium	−5.31	−5.37	−0.06	4.39	8.03
Butylammonium	−5.35	−5.37	−0.02	4.39	7.61
Cyclopentylammonium	−5.30	−5.39	−0.10	4.39	7.61
4-ammonium butyric acid	−5.55	−5.37	0.17	4.39	5.72
Pentylammonium	−5.29	−5.38	−0.09	4.38	6.96

Continued on next page

Table S-7 – continued from previous page

Molecule	E_{int}	E_{ad}	E_{def}	$E_g^{mol/PVK}$	E_g^{mol}
Cyclohexylammonium	–5.24	–5.48	–0.24	4.38	7.45
5-hydroxypentylammonium	–5.40	–5.30	0.10	4.41	4.72
2-(2-thienyl)ethanaminium	–5.25	–5.39	–0.14	4.34	5.44
Cyclohexylmethylammonium	–5.25	–5.39	–0.14	4.39	6.47
Hexylammonium	–5.28	–5.38	–0.10	4.39	6.38
1-methyl-hexylammonium	–5.24	–5.51	–0.27	4.38	7.14
1-methyl-butylammonium	–5.21	–5.57	–0.36	4.39	8.13
1-methyl-propylammonium	–5.26	–5.41	–0.15	4.41	8.25
Methylhydrazinium	–5.53	–5.28	0.25	4.41	7.31
4-amino-1-butyne protonated	–5.31	–5.41	–0.10	4.39	6.78
2-fluoroethylammonium	–5.51	–5.31	0.20	4.38	8.94
2-(2-thienyl)methylaminium	–5.25	–5.18	0.07	4.14	5.08
Isopropylammonium	–5.30	–5.38	–0.08	4.40	9.20
2-hydroxyethylammonium	–5.76	–5.47	0.28	4.32	7.58
Allyammonium	–5.34	–5.07	0.27	4.36	6.44
3,3-difluorocyclohexylammonium	–5.17	–5.53	–0.36	4.37	7.83
Anilinium	–5.41	–5.45	–0.04	4.12	6.09
5-valeric ammonium acid	–4.93	–5.38	–0.44	3.93	7.58
**Mean**	–5.34	–5.37	–0.03	4.35	7.32

Table S-8. Molecules assigned to Group 2 and their energetic/electronic descriptors (E_{int}, E_{ad}, E_{def}, $E_g^{mol/PVK}$, and E_g^{mol}). Energies are in eV.

Molecule	E_{int}	E_{ad}	E_{def}	$E_g^{mol/PVK}$	E_g^{mol}
Phenylmethylammonium	–5.22	–5.16	0.06	4.15	5.41
3-(dimethylamino)-1-propylammonium	–5.34	–5.39	–0.05	4.39	3.97
4-methylbenzylammonium	–5.14	–5.19	–0.05	4.27	5.11
Phenylethylammonium	–5.26	–5.46	–0.21	4.33	5.58
O-fluorophenylethylammonium	–5.16	–5.36	–0.20	4.12	5.55
M-fluorophenylethylammonium	–5.44	–5.33	0.12	4.19	4.45
P-fluorophenylethylammonium	–5.43	–5.33	0.10	4.18	4.43
2-ethyl-hexylammonium	–5.28	–5.39	–0.11	4.35	6.21
Octylammonium	–5.33	–5.41	–0.07	4.40	5.76
Nonylammonium	–5.26	–5.39	–0.13	4.39	5.28
Naphthalene-O-ethylammonium	–5.60	–5.31	0.29	3.92	2.88
Decylammonium	–5.25	–5.29	–0.03	4.39	4.80
4-fluorophenylmethylammonium	–5.35	–5.19	0.16	4.12	5.30
Heptylammonium	–5.27	–5.37	–0.10	4.40	5.94
Cycloheptylammonium	–5.15	–5.41	–0.26	4.39	6.91
Cyclooctylammonium	–5.15	–5.46	–0.31	4.40	6.49
Phenylpropylammonium	–5.29	–5.26	0.04	4.40	4.01
Phenylbutylammonium	–5.31	–5.30	0.01	4.40	3.66
1-(2-naphthyl)- methanammonium	–5.16	–5.01	0.15	3.45	3.92
2-(2-naphthyl)- ethanammonium	–5.31	–5.31	–0.01	3.56	3.35

Continued on next page

Table S-8 – continued from previous page

Molecule	E_{int}	E_{ad}	E_{def}	$E_g^{mol/PVK}$	E_g^{mol}
Naphthalene-O-propylammonium	−5.46	−5.41	0.05	3.96	2.90
N-dodecylammonium	−5.25	−5.37	−0.12	4.38	4.77
2-naphthyleneethylammonium	−5.21	−5.38	−0.17	3.59	4.18
2-(1-cyclohexenyl)ethylammonium	−5.25	−5.31	−0.06	4.35	4.47
P-methylphenylethylammonium	−5.30	−5.31	−0.01	4.35	4.22
P-biphenylamine	−5.31	−5.33	−0.02	3.61	4.14
R/S/1-phenylethylammonium	−5.18	−5.36	−0.18	4.29	5.61
Tetradecan-1-aminium	−5.27	−5.29	−0.02	4.40	4.13
Hexadecylammonium	−5.25	−5.29	−0.05	4.41	4.14
1-adamantylmethyazanium	−5.21	−5.40	−0.19	4.38	5.78
**Mean**	−5.28	−5.33	−0.05	4.20	4.78

Table S-9. Molecules assigned to Group 3 and their energetic/electronic descriptors (E_{int}, E_{ad}, E_{def}, $E_g^{mol/PVK}$, and E_g^{mol}). Energies are in eV.

Molecule	E_{int}	E_{ad}	E_{def}	$E_g^{mol/PVK}$	E_g^{mol}
2-(dimethylamino)ethylammonium	−4.01	−5.31	−1.30	4.17	7.70
Tetrametilamônio	−4.02	−5.17	−1.15	4.25	11.24
Tetraetilamônio	−3.92	−5.63	−1.71	4.38	9.98
Tetrapropilamônio	−3.84	−5.66	−1.81	4.31	9.26
Tetrabutilamônio	−3.90	−5.75	−1.85	4.32	8.74
Trimetiletilamônio	−3.96	−5.28	−1.32	4.29	10.09
Dimetildietilamônio	−3.94	−5.36	−1.42	4.32	10.09
Metiltrietilamônio	−3.97	−5.53	−1.56	4.38	9.79
Trimetilpropilamônio	−3.93	−5.31	−1.39	4.32	9.11
Trimetilbutilamônio	−3.91	−5.31	−1.39	4.33	8.34
Benziltrimetilamônio	−3.83	−4.74	−0.91	2.94	5.69
(2-Metoxietil)trimetilamônio	−4.03	−5.30	−1.27	4.33	6.79
N-Metil-N-propilpirrolidínio	−4.07	−5.51	−1.44	4.34	9.25
N-Butil-N-metilpirrolidínio	−3.94	−5.46	−1.53	4.34	8.45
N-Metil-N-pentilpirrolidínio	−4.05	−5.57	−1.52	4.34	7.81
N-Hexil-N-metilpirrolidínio	−4.08	−5.56	−1.48	4.35	7.69
N,N-Dietilpirrolidínio	−3.97	−5.56	−1.58	4.35	9.18
N-Propil-N-etilpirrolidínio	−3.98	−5.52	−1.54	4.33	9.03
N-Butil-N-etilpirrolidínio	−4.06	−5.61	−1.55	4.28	8.57
N-Metil-N-propilpiperidínio	−3.99	−5.60	−1.60	4.35	8.96
N-Butil-N-metilpiperidínio	−3.99	−5.63	−1.63	4.35	8.59
N-Metil-N-pentilpiperidínio	−3.97	−5.64	−1.67	4.33	8.29
N,N-Dietilpiperidínio	−4.02	−5.71	−1.69	4.35	9.08
N-Propil-N-etilpiperidínio	−3.93	−5.61	−1.67	4.35	8.83
Acetilcolina	−3.97	−5.14	−1.17	3.72	6.70
**Mean**	−3.97	−5.46	−1.49	4.24	8.69

Table S-10. Molecules assigned to Group 4 and their energetic/electronic descriptors (E_{int}, E_{ad}, E_{def}, $E_g^{mol/PVK}$, and E_g^{mol}). Energies are in eV.

Molecule	E_{int}	E_{ad}	E_{def}	$E_g^{mol/PVK}$	E_g^{mol}
3-iodopyridinium	−4.61	−3.03	1.58	2.06	3.92
4-amidinopyridinium	−4.43	−3.09	1.34	2.17	3.50
3-amidinopyridinium	−4.46	−2.92	1.54	1.93	3.34
N-Etilpiridínio	−4.06	−3.02	1.04	2.10	5.56
N-Propilpiridínio	−4.03	−3.02	1.01	2.12	5.54
N-Butilpiridínio	−4.14	−3.14	0.99	2.39	5.32
N-Hexilpiridínio	−4.13	−3.16	0.97	2.38	4.40
1-Butil-3-metilpiridínio	−4.14	−3.24	0.90	2.55	5.18
1-Butil-4-metilpiridínio	−4.07	−3.34	0.73	2.44	5.42
**Mean**	−4.23	−3.11	1.12	2.24	4.69

Table S-11. Molecules assigned to Group 5 and their energetic/electronic descriptors (E_{int}, E_{ad}, E_{def}, $E_g^{mol/PVK}$, and E_g^{mol}). Energies are in eV.

Molecule	E_{int}	E_{ad}	E_{def}	$E_g^{mol/PVK}$	E_g^{mol}
2-bromoethylammonium	−5.46	−5.40	0.06	4.34	6.82
3-bromopropylammonium	−5.16	−5.44	−0.29	4.29	6.88
4-bromobutylammonium	−5.54	−5.29	0.25	4.38	5.35
5-bromoxypentylammonium	−5.38	−5.30	0.08	4.39	4.44
2-bromphenylethylammonium	−5.32	−5.44	−0.12	4.28	5.15
4-bromophenylmethylammonium	−5.34	−5.09	0.25	3.92	4.59
P-bromphenylethylammonium	−5.47	−5.52	−0.05	4.26	4.81
**Mean**	−5.38	−5.35	0.03	4.27	5.44

Table S-12. Molecules assigned to Group 6 and their energetic/electronic descriptors (E_{int}, E_{ad}, E_{def}, $E_g^{mol/PVK}$, and E_g^{mol}). Energies are in eV.

Molecule	E_{int}	E_{ad}	E_{def}	$E_g^{mol/PVK}$	E_g^{mol}
3-iodopropylammonium	−5.47	−5.29	0.18	4.00	4.74
4-iodobutylammonium	−5.54	−5.42	0.13	4.31	4.32
6-iodohexylammonium	−5.40	−5.27	0.13	4.23	3.44
5-iodopentylammonium	−5.43	−5.43	0.01	4.24	4.31
4-iodophenylmethylammonium	−5.35	−5.09	0.26	3.95	4.10
4-amino-1,2-diiodo-1-butene protonated	−5.49	−5.31	0.18	3.50	4.03
**Mean**	−5.45	−5.30	0.15	4.04	4.16

Table S-13. Molecules assigned to Group 7 and their energetic/electronic descriptors (E_{int}, E_{ad}, E_{def}, $E_g^{mol/PVK}$, and E_g^{mol}). Energies are in eV.

Molecule	E_{int}	E_{ad}	E_{def}	$E_g^{mol/PVK}$	E_g^{mol}
Protonated 2-(aminoethyl)isothiourea	–4.99	–5.83	–0.84	4.30	6.47
1,2,4-triazolium	–5.14	–4.35	0.79	3.58	7.03
Formamidinuim	–5.22	–4.65	0.57	3.85	7.24
N,N,N′,N′-Tetrametilguanidínio	–4.47	–5.46	–0.99	4.38	6.30
Guanidinium	–5.55	–5.59	–0.05	4.28	8.03
**Mean**	–5.07	–5.18	–0.10	4.08	7.01

Table S-14. Molecules assigned to Group 8 and their energetic/electronic descriptors (E_{int}, E_{ad}, E_{def}, $E_g^{mol/PVK}$, and E_g^{mol}). Energies are in eV.

Molecule	E_{int}	E_{ad}	E_{def}	$E_g^{mol/PVK}$	E_g^{mol}
4-chlorophenylmethylammonium	–5.36	–5.10	0.25	3.97	4.85
2-chloroethylammonium	–5.47	–5.40	0.07	4.37	7.48
P-Chloroanilinium	–5.56	–5.45	0.11	3.91	5.34
P-chlorophenylethylammonium	–5.41	–5.45	–0.04	4.29	5.03
R/S/rac-1-(4-chlorophenyl)ethylammonium	–5.34	–5.34	0.00	4.11	5.04
**Mean**	–5.43	–5.35	0.08	4.13	5.55

Table S-15. Molecules assigned to Group 9 and their energetic/electronic descriptors (E_{int}, E_{ad}, E_{def}, $E_g^{mol/PVK}$, and E_g^{mol}). Energies are in eV.

Molecule	E_{int}	E_{ad}	E_{def}	$E_g^{mol/PVK}$	E_g^{mol}
Cyclohexylphosphonium	–4.83	–5.34	–0.51	4.35	7.31
Tetrametilfosfônio	–4.06	–5.29	–1.23	4.33	10.43
Tetraetilfosfônio	–3.87	–5.53	–1.67	4.32	9.40
Tetrabutilfosfônio	–4.02	–5.75	–1.74	4.34	8.41
**Mean**	–4.19	–5.48	–1.29	4.34	8.89

Table S-16. Molecules assigned to Group 10 and their energetic/electronic descriptors (E_{int}, E_{ad}, E_{def}, $E_g^{mol/PVK}$, and E_g^{mol}). Energies are in eV.

Molecule	E_{int}	E_{ad}	E_{def}	$E_g^{mol/PVK}$	E_g^{mol}
Pyrene-O-ethylammonium	–5.40	–4.62	0.78	1.77	1.75
Pyrene-O-propylammonium	–5.99	–4.42	1.57	1.81	1.74
Pyrene-O-buthylammonium	–5.34	–4.95	0.39	1.78	1.63

Continued on next page

Table S-16 – continued from previous page

Molecule	E_{int}	E_{ad}	E_{def}	$E_g^{mol/PVK}$	E_g^{mol}
Pyrene-butylammonium	−5.32	−4.86	0.47	1.79	1.61
**Mean**	−5.51	−4.71	0.80	1.79	1.68

Table S-17. Molecules assigned to Group 11 and their energetic/electronic descriptors (E_{int}, E_{ad}, E_{def}, $E_g^{mol/PVK}$, and E_g^{mol}). Energies are in eV.

Molecule	E_{int}	E_{ad}	E_{def}	$E_g^{mol/PVK}$	E_g^{mol}
2,3,4,5,6-pentafluorophenethylammonium	−5.61	−5.27	0.34	3.80	4.71
2,2-difluoroethylammonium	−5.83	−5.30	0.54	4.34	8.74
2,2,2-trifluoroethylammonium	−5.83	−5.26	0.57	4.36	9.88
Perfluorobenzylammonium	−5.67	−5.04	0.64	3.87	5.20
**Mean**	−5.74	−5.21	0.52	4.09	7.13

Table S-18. Molecules assigned to Group 12 and their energetic/electronic descriptors (E_{int}, E_{ad}, E_{def}, $E_g^{mol/PVK}$, and E_g^{mol}). Energies are in eV.

Molecule	E_{int}	E_{ad}	E_{def}	$E_g^{mol/PVK}$	E_g^{mol}
trimethylsulfonium	−4.68	−5.47	−0.79	4.39	8.74
Trietilsulfônio	−4.35	−5.36	−1.01	4.36	8.32
Dietilmetilsulfônio	−4.47	−5.42	−0.95	4.35	8.45
**Mean**	−4.50	−5.42	−0.92	4.37	8.50

Table S-19. Molecules assigned to Group 13 and their energetic/electronic descriptors (E_{int}, E_{ad}, E_{def}, $E_g^{mol/PVK}$, and E_g^{mol}). Energies are in eV.

Molecule	E_{int}	E_{ad}	E_{def}	$E_g^{mol/PVK}$	E_g^{mol}
Trihexiltetradecilfosfônio	−4.03	−5.88	−1.85	4.37	6.13
**Mean**	−4.03	−5.88	−1.85	4.37	6.13

References

1 Ribeiro, I. C.; Moraes, P. I. R.; Bittencourt, A. F.; Da Silva, J. L. Unveiling the impact of organic cation passivation on structural and optoelectronic properties of two-dimensional perovskites thin films. *Applied Surface Science* **2024**, *678*, 161098, DOI: `10.1016/j.apsusc.2024.161098`.

2 Hirshfeld, F. L. Bonded-Atom Fragments for Describing Molecular Charge Densities. *Theor. Chim. Acta* **1977**, *44*, 129–138, DOI: 10.1007/bf00549096.

3 Lloyd, S. Least Squares Quantization in PCM. *IEEE Trans. Inform. Theory* **1982**, *28*, 129–137, DOI: 10.1109/tit.1982.1056489.

4 Pedregosa, F.; Varoquaux, G.; Gramfort, A.; Michel, V.; Thirion, B.; Grisel, O.; Blondel, M.; Prettenhofer, P.; Weiss, R.; Dubourg, V.; Vanderplas, J.; Passos, A.; Cournapeau, D.; Brucher, M.; Perrot, M.; Duchesnay, E. Scikit-learn: Machine Learning in Python. *Journal of Machine Learning Research* **2011**, *12*, 2825–2830.

5 Ward, J. H. Hierarchical Grouping to Optimize an Objective Function. *Journal of the American Statistical Association* **1963**, *58*, 236–244.
